# Stathmin 1/2-triggered microtubule loss mediates Golgi fragmentation in mutant SOD1 motor neurons

**DOI:** 10.1186/s13024-016-0111-6

**Published:** 2016-06-09

**Authors:** Sarah Bellouze, Gilbert Baillat, Dorothée Buttigieg, Pierre de la Grange, Catherine Rabouille, Georg Haase

**Affiliations:** Institut de Neurosciences de la Timone, UMR 7289, Centre National de la Recherche Scientifique (CNRS) and Aix-Marseille Université, 27 bd Jean Moulin, 13005 Marseille, France; GenoSplice technology, iPEPS - ICM, Hôpital Pitié Salpêtrière, 47/83, bd de l’Hôpital, 75013 Paris, France; Department of Cell Biology, Hubrecht Institute of the KNAW & UMC Utrecht, Uppsalalaan 8, 3584 CT Utrecht, Netherlands

## Abstract

**Background:**

Pathological Golgi fragmentation represents a constant pre-clinical feature of many neurodegenerative diseases including amyotrophic lateral sclerosis (ALS) but its molecular mechanisms remain hitherto unclear.

**Results:**

Here, we show that the severe Golgi fragmentation in transgenic mutant SOD1^G85R^ and SOD1^G93A^ mouse motor neurons is associated with defective polymerization of Golgi-derived microtubules, loss of the COPI coat subunit β-COP, cytoplasmic dispersion of the Golgi tether GM130, strong accumulation of the ER-Golgi v-SNAREs GS15 and GS28 as well as tubular/vesicular Golgi fragmentation. Data mining, transcriptomic and protein analyses demonstrate that both SOD1 mutants cause early presymptomatic and rapidly progressive up-regulation of the microtubule-destabilizing proteins Stathmins 1 and 2. Remarkably, mutant SOD1-triggered Golgi fragmentation and Golgi SNARE accumulation are recapitulated by Stathmin 1/2 overexpression but completely rescued by Stathmin 1/2 knockdown or the microtubule-stabilizing drug Taxol.

**Conclusions:**

We conclude that Stathmin-triggered microtubule destabilization mediates Golgi fragmentation in mutant SOD1-linked ALS and potentially also in related motor neuron diseases.

**Electronic supplementary material:**

The online version of this article (doi:10.1186/s13024-016-0111-6) contains supplementary material, which is available to authorized users.

## Background

Structural alterations of the Golgi apparatus are among the earliest and most constant pathological features in neurodegenerative diseases and have been widely studied in the motor neuron disease amyotrophic lateral sclerosis (ALS) [16, 23, 57]. The Golgi alterations are detectable in degenerating ALS motor neurons of spinal cord and cerebral motor cortex [20, 36], are common to both sporadic [22] and familial forms of the disease [21, 24, 31, 36, 58], and manifest at presymptomatic stage [36, 65, 66].

The Golgi apparatus of normal motor neurons is made of stacked membrane-bound cisternae that are laterally connected to form the Golgi ribbon [5]. Earlier studies in particular by Gonatas and colleagues have characterized the structural Golgi alterations in ALS motor neurons as fragmentation [22], i.e. transformation of the Golgi ribbon into disconnected stacks or into tubules and vesicles [5], and as atrophy [22, 36], i.e. loss or dispersion of Golgi membranes. In the most widely studied ALS mouse model, transgenic mutant SOD1^G93A^ mice, Golgi fragmentation can affect up to 75 % of spinal cord motor neurons at symptomatic stage [36, 65].

The Golgi apparatus is a highly dynamic cellular organelle that ensures the processing and sorting of proteins form their site of synthesis in the endoplasmic reticulum (ER) en route to their final destination, which is reflected by its functional division into a cis(entry) side and a trans(exit) side. Intra-Golgi transport involves COPI-coated vesicles [2] which are formed through recruitment of coatomers α-ζ [43], tethered by Rabs and Golgins [37] and fused/docked to target membranes by Golgi SNAREs [29].

While the molecular mechanisms of Golgi fragmentation in ALS remain largely to be deciphered, at least two mechanisms can been proposed. The first mechanism involves an impairment in transport from endoplasmic reticulum (ER) to Golgi [4, 52] and from Golgi to plasma membrane [54]. Both could in turn affect Golgi structure. The second mechanism involves potential microtubule alterations [36]. Microtubules are indeed closely associated with the Golgi [30, 50] and nucleated at its membrane [8, 14] in motor neurons [5]. Furthermore, pharmacological microtubule disruption with colchicine or nocodazole causes reversible Golgi fragmentation and dispersal [15, 47, 61]. Finally, we have recently demonstrated that defective polymerization of Golgi-derived microtubules causes Golgi fragmentation in motor neurons of *pmn* mice with progressive motor neuronopathy which are mutated in the tubulin-binding cofactor E (Tbce) gene [5].

The role of microtubules in mutant SOD1-linked Golgi fragmentation remained however unclear. Indeed, microtubules appeared normal in motor neuron cell bodies with a fragmented Golgi [53] despite their early alterations in axons [17, 70]. In addition, dys-regulation of a microtubule-severing protein (Stathmin-1) was seen only in a fraction of motor neurons with Golgi fragmentation and only at late disease stage [55], suggesting that it may represent a compensatory event.

To analyze the mechanisms of Golgi pathology in ALS, we here investigated two transgenic mouse lines with similar disease course due to either dismutase active (G93A) or inactive (G85R) human SOD1. Mutant SOD1^G93A^ mice (line G1del) develop limb weakness between day 165 to 240 and fatal paralysis about 40 days later [1]. Mutant SOD1^G85R^ mice (line 148) develop limb weakness between day 240 to 280 and die about 15 days later [6]. As controls, we used non-transgenic litter mice and mice expressing wild type human SOD1 (SOD1^wt^, line 76) at a similar level as in the mutants. In addition, we used NSC-34 cells transfected with mutant or wildtype SOD1 as in vitro system. We show that the early Golgi pathology observed in mutant SOD1^G85R^ and SOD1^G93A^ motor neurons involves disruption of the Golgi-nucleated somatic microtubule network due to up-regulation of the two microtubule-severing proteins Stathmin 1 and 2. This in turn leads to Golgi fragmentation with early pre-symptomatic and rapidly progressive accumulation of the ER-Golgi v-SNAREs GS28 and GS15. These data, together with our findings in ALS-like *pmn* mice [5], lead us to propose that a disruption of Golgi-nucleated microtubules underlies Golgi fragmentation in human SOD1-linked ALS and related degenerative motor neuron diseases.

## Results

**Fig. 1:**
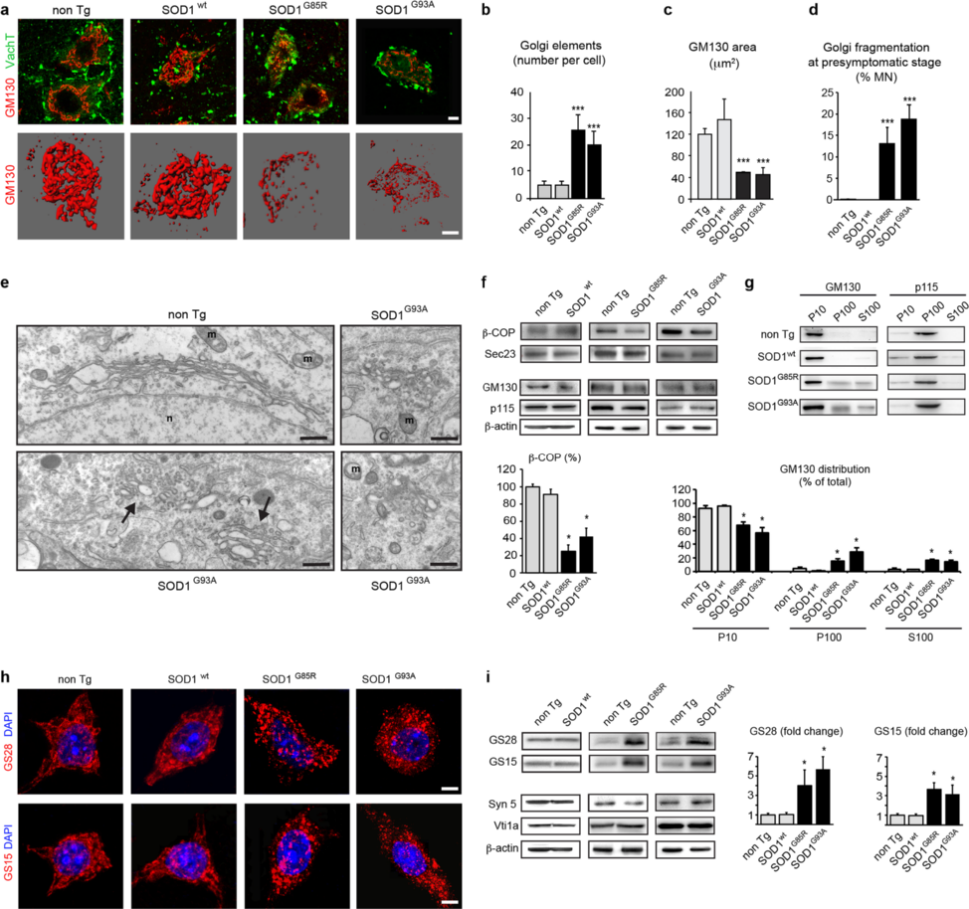
Morphological and molecular Golgi alterations in motor neurons of mutant SOD1 mice. **a**. Confocal z-stacks (upper panels) show fragmentation of GM130-labeled Golgi membranes in lumbar motor neurons of 240-day-old mutant SOD1^G85R^ and SOD1^G93A^ mice as compared to non-transgenic mice and transgenic SOD1^wt^ mice. Motor neurons are identified by expression of VaChT (vesicular acetylcholine transporter). 3D-modeling (lower panels) of GM130-labelled Golgi membranes in entire motor neurons confirms Golgi fragmentation. Scale bars 10 μm. **b**. Increased number of GM130-stained Golgi elements in mutant SOD1^G85R^ and SOD1^G93A^ motor neurons determined by 3D modeling of Golgi membranes in entire cells (mean ± sd, n = 12 motor neurons from three mice per genotype, *** p < 0.0001 by student’s *t*-test, unpaired. **c**. Decreased cross-sectional area of GM130-labeled Golgi area in mutant SOD1^G85R^ and SOD1^G93A^ motor neurons as compared to control motor neurons (mean ± sd, *** p < 0.001, n = 50 motor neurons from three mice per genotype, student's *t* test). See also Additional file 1: Figure S1. **d**. Percentage of motor neurons with GM130-labeled Golgi fragmentation at presymptomatic stage (mean ± sd, *** p < 0.001 by student’s *t*-test, n > 250 motor neurons from four mice per genotype were analyzed at presymptomatic stage corresponding to age 130 days (SOD1^G93A^, non Tg) or 180 days (SOD1^G85R^, SOD1^wt^). **e**. Electron microscopy of a lumbar motor neuron from a non-transgenic mouse aged 140 days showing a typical Golgi apparatus (upper panel) that is easily distinguished from unlinked, partially swollen and vesiculated Golgi profiles observed in mutant SOD1^G93A^ motor neurons (arrows in lower panel, magnifications on the right). n: nucleus, m: mitochondria. Scale bars 500 nm (left panels), 200 nm (right panels). **f**. Western blots showing decreased levels of β-COP in mutant SOD1 mice, and normal levels of Sec23, GM130 and p115. Loading control β-actin. Shown is one representative blot per genotype out of four. The diagram below shows that β-COP levels (normalized to β-actin) are reduced to 25 ± 7.7 % and 42.5 ± 9.6 % of non Tg (mean ± sd, n = 4 per genotype, * p < 0.01 by Mann Whitney test). **g**. Subcellular fractionation of spinal cords. Western blot analyses show redistribution of GM130 in mutant SOD1 mice, as indicated by shift from its normal membrane localization in control and SOD^wt^ mice into fragmented membranes and vesicles in SOD1^G85R^ and SOD1^G93A^ mice and cytosol. P115 is not redistributed. Each blot is representative of three independant experiments on mice of the indicated genotypes. The diagram below shows densitometric determination of protein distribution (mean ± sd, n = 3 per genotype, * p < 0.01 by Mann Whitney test). **h**. Confocal imaging reveals accumulation of Golgi v-SNARE protein GS28 (upper panels) and GS15 (lower panels) in small vesicle-like punctae of motor neurons in mutant SOD1 mice, as compared to controls. Scale bars 10 μm. **i**. Western blots (upper two panels) demonstrating massively increased levels of Golgi v-SNAREs GS28 and GS15 in mutant SOD1^G85R^ and SOD1^G93A^ lumbar spinal cords, as compared to non-transgenic and SOD1^wt^ spinal cords. Western blots (lower three panels) showing normal expression of the Golgi t-SNARE Syntaxin-5a and the endosomal v-SNARE Vti1a. β-actin indicates equal protein loading. Each blot is representative of three independant experiments on mice of the indicated genotypes. The diagrams show increased spinal cord protein levels of GS28 by 4 ± 1.6 (SOD1^G85R^) and by 3.7 ± 1.3 (SOD1^G93A^) fold and of GS15 by 3.7 ± 0.7 (SOD1^G85R^) and by 3.2 ± 1 (SOD1^G93A^) as compared to non Tg control (mean ± sd, * p < 0.001 by Mann Whitney test)

To confirm the reported Golgi alterations in mutant SOD1 motor neurons [36, 65, 66], we first examined lumbar spinal cord cryosections from 240-day-old mice by confocal microscopy using GM130 antibodies. We demonstrate that Golgi membranes form a dense ribbon in the cell bodies of control and SOD1^wt^ motor neurons, but appear fragmented or atrophied in motor neurons of mutant SOD1^G85R^ and SOD1^G93A^ mice (Fig. 1a). Golgi fragmentation was confirmed by GM130 membrane modeling demonstrating an ~4 fold increase in the number of individual Golgi profiles in SOD1^G85R^ and SOD1^G93A^ motor neurons when compared to control (Fig. 1a,b). Loss of Golgi area in SOD1^G85R^ and SOD1^G93A^ motor neurons was quantified by a reduction in its cross-sectional area to ~35 % of normal (Fig. 1a,c, Additional file 1: Figure S1A).

To confirm that Golgi fragmentation precedes disease onset in ALS [36, 65, 66], we analyzed mutant SOD1^G93A^ mice and SOD1^G85R^ mice, respectively one month before (age 130 days) and two months before (age 180 days) the onset of first clinical symptoms and before any reported loss of motor neurons. Up to 19 % of mutant SOD1 motor neurons displayed signs of Golgi fragmentation labelled by GM130, as compared to < 1 % of motor neurons in non-transgenic and SOD1^wt^ mice (Fig. 1d and Additional file 1: Figure S1B). This compares very well with published results at least in mutant SOD1^G93A^ mice, where Golgi fragmentation is scored in 25-30 % of motor neurons about 10 days later at 20 weeks of age [65].

To confirm and characterize the changes of the Golgi architecture, we analyzed presymptomatic mutant SOD1 mice aged 140 days at the ultrastructural level by electron microscopy. In control mice (Fig. 1e, upper left panel) and in transgenic SOD1^wt^ mice (not shown), the Golgi apparatus exhibits the typical morphology of stacked cisternae as described in [5]. Conversely, in motor neurons of mutant SOD1 mice, we observe a fragmentation of the Golgi ribbon into many disconnected Golgi stacks, small tubules and vesicles that appear uncoated as well as unlinked (Fig. 1e, arrows in lower left panels).

To analyze the molecular basis of the observed Golgi fragmentation in mutant SOD1 motor neurons, we investigated the status of Golgi vesicle coats, tethers and SNAREs using biochemical methods. We found that the level of the COPI vesicle subunit β-COP is lower in mutant SOD1^G85R^ and SOD1^G93A^ spinal cords than in control spinal cords, whereas the level of the COPII vesicle subunit Sec23 remains normal (Fig. 1f, upper panels).

The Golgi tethering protein GM130 is normally associated with the cytoplasmic face of cis-medial Golgi membranes and binds the Golgi tether p115 present on incoming vesicles [38]. We found that the total levels of GM130 and p115 are unchanged in mutant SOD1 spinal cords when compared to control (Fig. 1f, lower panels). Since the membrane association of GM130 and p115 is critical for their function [37], we investigated a potential subcellular redistribution of both Golgi tethers in mutant SOD1 mice by biochemical fractionation of spinal cords into membrane (P10), vesicle (P100) and cytosol (S100) fractions. We found that GM130 is significantly redistributed from its typical Golgi membrane localization in control mice to vesicles, membrane fragments and cytosol in SOD1^G85R^ and SOD1^G93A^ mice (Fig. 1g, blots and diagram). Interestingly, this subcellular redistribution is specific for GM130 since p115 remains associated with the vesicle fraction as in control mice (Fig. 1g). The purity of the P10, P100 and S100 fractions was confirmed by analysis of molecular markers L1, Vt1a and GAPDH (Additional file 1: Figure S2). These data suggest that mutant SOD1 expression leads to defective tethering of Golgi vesicles due to redistribution of GM130.

Last, Golgi SNAREs mediate vesicle fusion through interaction of v-SNAREs (present on vesicles) and t-SNAREs (present on target membranes) [26, 29]. Using immunofluorescence, we found that the ER-Golgi v-SNAREs GS28 [56] and GS15 [71] are present in small dispersed profiles in mutant SOD1^G85R^ and SOD1^G93A^ motor neurons when compared to control motor neurons (Fig. 1h). Furthermore, Western blots showed that the total spinal cord levels of GS28 and GS15 are increased by > 4 fold and >3 fold respectively in mutant SOD1^G85R^ mice and SOD1^G93A^ mice when compared to control mice (Fig. 1i, upper panels and diagrams). This is specific for GS15 and 28 because, by contrast, the levels of Syntaxin5a, the major Golgi t-SNARE cognate of GS28 and GS15, and of the endosomal v-SNARE Vti1a, are unchanged in mutant SOD1 mice (Fig. 1i, lower panels). Importantly in SOD1^wt^ spinal cords, the levels of all tested SNARE proteins are identical to those in non-transgenic mice (Fig. 1h, upper panels), indicating a specific and significant increase of Golgi v-SNAREs GS28 and GS15 in mutant SOD1 spinal cords.

**Fig. 2:**
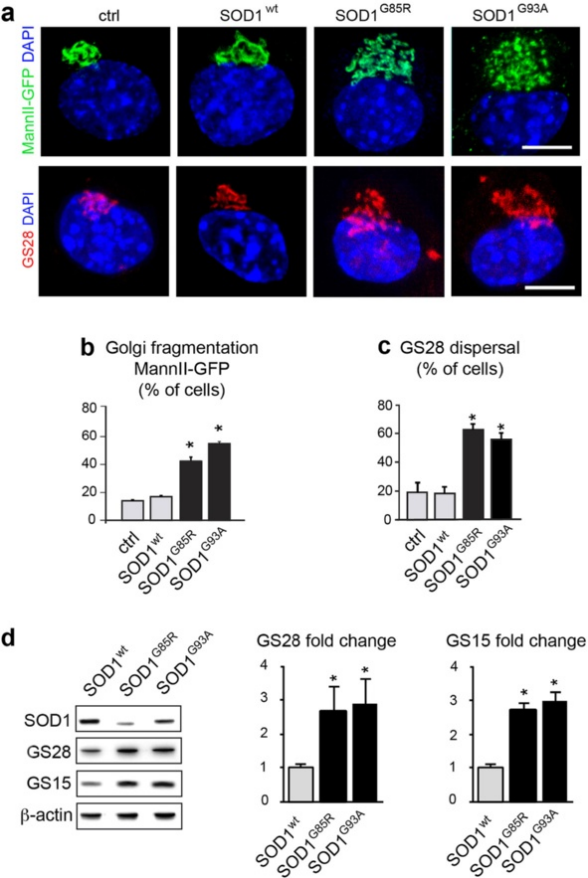
Golgi alterations in mutant SOD1-transfected NSC-34 motor neurons. **a**. Confocal images (upper panels) showing Golgi fragmentation with the marker MannII-GFP in NSC-34 cells transfected for 4 DIV with mutant SOD1, as compared to the situation in control or wildtype SOD1-transfected cells. Confocal images (lower panels) showing GS28 dispersal after transfection with mutant SOD1, as compared to the situation in control or wildtype SOD1 cells. Scale bar 10 μm. **b**. Percentage of cells (mean ± sd) with Golgi fragmentation labeled for MannII-GFP. More than 300 cells per condition were analyzed on quadruplicate wells per condition. Statistical significance * p < 0.01 by Mann–Whitney test, as compared to mock and SOD^wt^. Data represent one out of three experiments yielding similar results. **c**. Percentage of transfected cells with dispersal of GS28. More than 200 cells per condition were analyzed on quadruplicate wells per condition. Statistical significance * p < 0.01 by Mann–Whitney test, as compared to mock and SOD^wt^. **d**. Western blot analysis of NSC-34 cells demonstrating that GS28 protein levels are increased to 2.7 ± 0.7 and 2.9 ± 0.8 by SOD1^G85R^ and SOD1^G93A^, respectively, as compared to those in control SOD^wt^. GS15 protein levels are increased to 2.7 ± 0.2 and 3.0 ± 0.3 fold, respectively. The diagrams show densitometric quantification of protein levels relative to SOD^wt^ (mean ± sd). Cellular extracts from three independently transfected cultures were blotted and analyzed each in duplicate. Statistical significance * p < 0.01 by Mann Whitney test

In order to set up a tractable in vitro model, we reproduced these Golgi alterations in cultured NSC-34 motor neurons [7] expressing mutant SOD1 (Fig. 2). Using MannII-GFP or GM130 as Golgi markers, the Golgi appears as a compact juxtanuclear structure in control cells transfected with empty or SOD1^wt^ plasmids but is fragmented in cells transfected with SOD1^G85R^ or SOD1^G93A^ plasmids (Fig. 2a,b and Additional file 1: Figure S1C). Expression of both SOD1 mutants also triggers dispersal of GS28, as shown by IF (Fig. 2a,c) and a >2 fold increase in protein levels of GS28 and GS15, as shown by Western blot (Fig. 2d).

Taken together, these results show that both in spinal motor neurons and in NSC-34 cells, expression of mutant SOD1^G85R^ and SOD1^G93A^ causes loss of β-COP, re-distribution of GM130 and strong accumulation of Golgi SNAREs GS28 and GS15, in line with the observed Golgi fragmentation. Importantly, these alterations closely resemble those that we previously observed in *pmn* mice [5].

### Mutant SOD1 proteins localize to the Golgi and impede the polymerization of Golgi-derived microtubules

Given that the molecular features observed in *pmn* mice are triggered by a point mutation in Golgi-localized TBCE that affects the Golgi microtubule network, we wondered whether mutant SOD1 also impairs Golgi-derived microtubules.

**Fig. 3:**
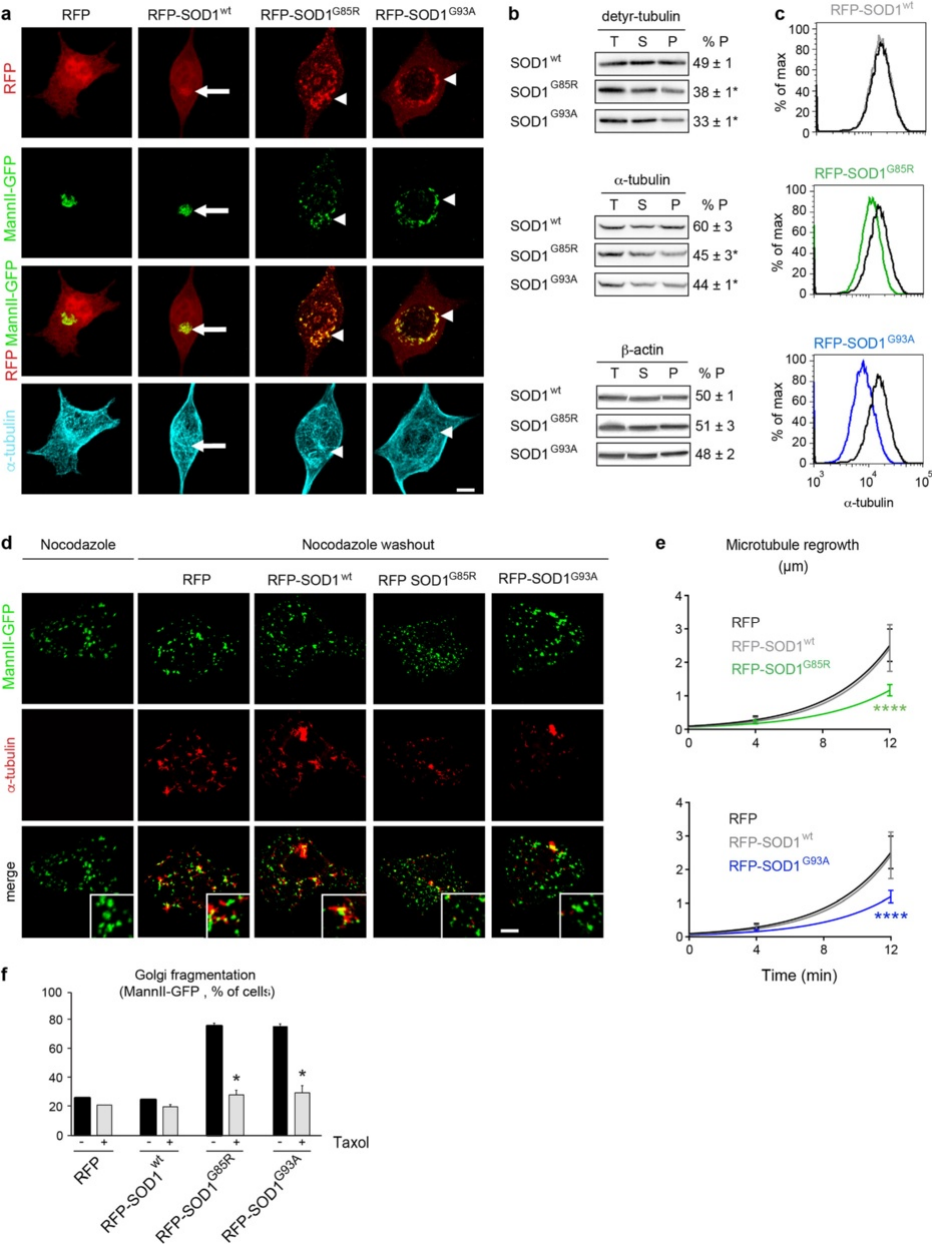
SOD1 mutants impair polymerization of Golgi-derived microtubules. **a**. Images show NSC-34 cells expressing RFP or SOD1 variants tagged with RFP after extraction for soluble proteins and labeling with MannII-GFP (Golgi) and antibodies against α-tubulin (microtubules). Merged RFP/MannII-GFP images show that both wildtype SOD1 (arrow) and mutant SOD1 (arrowheads) localize to Golgi membranes. This is confirmed by Pearson’s correlation analysis (RFP-SOD1^wt^ 0.74 ± 0.09, RFP-SOD1^G85R^ 0.72 ± 0.09, RFP-SOD1^G93A^ 0.77 ± 0.14, RFP 0.13 ± 0.14, statistical significance SOD1 variants vs RFP : p < 0.0006 by student’s *t*-test). Mutant SOD1^G85R^ and SOD1^G93A^ specifically cause rarefaction of microtubules around fragmented Golgi profiles (arrowheads in lower panel). **b**. Biochemical fractionation of total (T), soluble (S) and polymerized (P) tubulins. Cells expressing SOD1^G85R^ or SOD1^G93A^ display a decreased ratio of polymerized detyrosinated (detyr-) tubulin. The ratio of polymerized α-tubulin is also decreased. Polymerization of β-actin is not affected by mutant SOD1. % P is equivalent to P/(P + S), * p < 0.01 by Mann–Whitney test, n = 3 experiments each. **c**. Flow cytometry of cellular microtubules in NSC-34 cells after extraction of soluble tubulins, microtubule stabilization and intracellular labeling with α-tubulin-FITC antibodies*.* Cells expressing SOD^wt^ (in grey, upper panel) display normal levels of α-tubulin-containing microtubules in comparison to cells expressing RFP (in black). Cells expressing mutant SOD1^G85R^ (in green, middle panel) or mutant SOD1^G93A^ (in blue, lower panel) show decreased levels of cellular microtubules. Median fluorescence signal per cell: 15.800 (RFP), 15.900 (SOD1wt), 11.600 (SOD1^G85R^), 8.418 (SOD1^G93A^). Statistical significance by chi square test, * T(x) > 200, ns T(x) = 0. **d**. Images showing transfected NSC-34 cells that were treated with Nocodazole (left column) and restored to drug-free medium for 12 min (right four columns). Cells were identified by RFP expression (not shown), Golgi profiles were identified by MannII-GFP and growing microtubules with antibodies against α-tubulin (pseudocolored in red). Mutant SOD1^G85R^ and SOD1^G93A^ impede regrowth of Golgi-derived microtubules (zoomed insets). Scale bar 5 μm. **e**. Diagrams showing reduced growth rate of Golgi-derived microtubules in cells expressing RFP- SOD1^G85R^ (upper panel) or RFP- SOD1^G93A^ (lower panel) as compared to cells expressing RFP- SOD1^wt^ or RFP. Microtubule length represents mean of mean of >12 cells per time point and condition and a total of 1495 microtubules analyzed. Statistical significance **** p < 0.0001 (RFP-SOD1^G85R^ vs RFP or vs SOD1^wt^) and **** p < 0.0001 (RFP-SOD1^G93A^ vs RFP or vs SOD1^wt^) by ANOVA test and Tukey’s multiple comparison test. **f**. Diagrams showing Taxol-mediated rescue of MannII-GFP-labeled Golgi fragmentation in cells expressing mutant SOD1^G85R^ or SOD1^G93A^ each tagged to RFP. Statistical significance * p < 0.01 (Taxol vs mock) by Mann–Whitney test, n ≥ 50 cells per well and 4 replicate wells were analyzed per condition

Previous studies in non-motor neuronal cells have localized a significant fraction of SOD1 to Golgi membranes [63, 64]. To confirm this, we transfected RFP-tagged forms of wildtype and mutant SOD1 together with MannII-GFP in NSC-34 cells. After extraction of soluble proteins, co-localization and Pearson correlation analysis showed that both RFP-tagged SOD1^wt^, SOD1^G85R^ and SOD1^G93A^ indeed significantly co-localize with MannII-GFP-labeled Golgi membranes whereas RFP is mainly nuclear (Fig. 3a, upper three panels).

Since the Golgi membrane is a major site of microtubule polymerization and nucleation in motor neurons [5, 8, 14], we then tested whether SOD1 mutants affect the polymerization of microtubules. By IF, microtubules appear disorganized and rarefied around MannII-GFP-labeled Golgi punctae in mutant SOD1 cells when compared to control cells (Fig. 3a, lower panel). To confirm this, we biochemically analyzed detyrosinated tubulins, which are markers of Golgi-associated microtubules [50, 60], by determining their total, soluble and polymerized fractions (Fig. 3b). The ratio of polymerized versus total detyrosinated tubulin is significantly lower in mutant SOD1^G85R^ cells and SOD1^G93A^ cells than in SOD1^wt^ cells (Fig. 3b, upper panels). The ratio of polymerized versus total α-tubulin in mutant SOD1 cells is also decreased, albeit to a lower extent (Fig. 3b, middle panels). These data indicate that both SOD1 mutants impede the polymerization of Golgi-derived microtubules.

To determine the microtubule content in individual cells, we extracted soluble tubulins, labeled polymerized microtubules with FITC-coupled antibodies against α-tubulin and performed flow cytometry. This method accurately measures cellular microtubules since the flow cytometry signals of cells are increased after treatment with the microtubule-stabilizing drug Taxol, decreased after treatement with the microtubule-disrupting drug Nocodazole and correlated with the amount of biochemically fractionated microtubules (Additional file 1: Figure S3A-B). Using this technique, we found significantly less polymerized microtubules in cells expressing RFP-tagged SOD1^G85R^ or SOD1^G93A^ than in cells expressing RFP-SOD1^wt^ or RFP (Fig. 3c), confirming the mutant SOD1-triggered loss of cellular microtubules.

To directly measure the polymerization rate of microtubules at the Golgi, we incubated control and mutant SOD1 NSC-34 cells with Nocodazole, restored the cells to drug-free medium and measured the regrowth of Golgi-derived microtubules over 12 min using α-tubulin/MannII-GFP labeling (Fig. 3d). In control cells expressing RFP or RFP-SOD1^wt^, small microtubule asters form at Golgi profiles within few minutes after nocodazole washout. These microtubules then grow rapidly, confirming the capacity of Golgi membranes to nucleate microtubules [5, 8, 14]. In mutant SOD1 expressing cells, however, Golgi-derived microtubule asters are rare and grow slowly. Their mean growth rate is reduced by over 50 % in SOD1^G85R^ and SOD1^G93A^ cells in comparison to RFP expressing cells (Fig. 3e). Wildtype SOD1 does not impede growth of Golgi-derived microtubules (Fig. 3e). Taken together, these data indicate that SOD1 mutants decrease the growth rate and steady state levels of Golgi-derived microtubules.

Last, to test whether these microtubule defects are responsible for the observed Golgi alterations, we stabilized microtubules in mutant SOD1- and control-transfected cells by treating the cultures with Taxol (10 nM) from 0 to 4 DIV. We found that Taxol significantly reduces the percentage of mutant SOD1-transfected cells harboring MannII-GFP-labeled Golgi alterations (Fig. 3f), indicating that microtubule defects mediate the mutant SOD1-triggered Golgi alterations.

### Mutant SOD1 causes up-regulation of Stathmin 1 and 2 gene expression

To identify the upstream triggers of microtubule loss and tubular/vesicular Golgi fragmentation in mutant SOD1 motor neurons, we performed data mining and bioinformatic analyses. We first analyzed five studies comparing the gene expression profiles of SOD1^G93A^ and control motor neurons isolated by laser capture microdissection from adult spinal cord [19, 28, 33, 40, 42]. Two studies showed that cytoskeleton-related genes annotated to the Gene Ontology (GO) terms “cytoskeletal part/GO:0044430” and “cytoskeleton/GO:0005856” are significantly dys-regulated: 14 genes at presymptomatic stage [33]), 76 genes at early disease stage [33]) and 42 genes at end stage [42]). Among those, genes annotated to the microtubule cytoskeleton (GO:0015630) display significant dys-regulation [33].

To identify individual dys-regulated genes linked to microtubules, we then compared by bioinformatics analysis the publically available gene expression profiles from mutant SOD1 G93A and control motor neurons deposited in the Gene Omnibus database by Ferraiuolo et al. as GSE10953 [19] and by Nardo et al. as GSE46298 [40]. Given the similarities between *pmn* and SOD1, we first focused on tubulin binding co-factors but found that the six genes Tbca, Tbcb, Tbcc, Tbcd, Tbce and Tbcel (Tbce-like) are not significantly dys-regulated in mutant SOD1 motor neurons at any time point (fold change < 1.5, p > 0.05, Table 1).

**Table 1:** Gene expression profiles of tubulin-binding cofactors in mutant SOD1 motor neurons

Ferraiuolo et al. 2007	Presymptomatic G93A vs. Ctrl (P60)			Symptomatic G93A vs. Ctrl (P90)			Endstage G93A vs. Ctrl (P120)		
Gene Symbol	Regulation	Fold-Change	*P*-Value	Regulation	Fold-Change	*P*-Value	Regulation	Fold-Change	*P*-Value
Tbca	up	1,74	1,61 E-01	up	1,13	6,11E-01	down	1,63	9,62E-02
Tbcb	up	1,03	9,24E-01	down	1,08	7,49E-01	down	1,33	6,17E-02
Tbcc	down	1,09	8,24E-01	down	1,25	4,57E-01	up	1,03	9,71E-01
Tbcd	down	1,14	8,10E-01	up	1,07	7,65E-01	down	1,03	9,32E-01
Tbce	up	1,29	6,22E-01	up	1,01	9,87E-01	down	1,07	9,18E-01
Tbcel	down	1,07	9,21 E-01	down	1,12	7,91 E-01	up	1,10	8,09E-01
Nardo et al. 2013	Presymptomatic C57 G93A vs. Ctrl			Onset C57 G93A vs. Ctrl			Symptomatic C57 G93A vs. Ctrl		
Gene	Regulation	Fold-Change	*P*-Value	Regulation	Fold-Change	*P*-Value	Regulation	Fold-Change	*P*-Value
Tbca	up	1,23	1,81 E-01	down	1,03	8,53E-01	up	1,04	3,83E-01
Tbcb	up	1,06	7,17E-01	down	1,15	5,21 E-01	down	1,04	8,46E-01
Tbcc	up	1,16	2,81 E-01	down	1,10	2,12E-01	up	1,07	6,83E-01
Tbcd	up	1,09	6,15E-01	up	1,01	9,47E-01	up	1,10	5,20E-01
Tbce	up	1,11	6,02E-01	up	1,17	2,70E-01	up	1,10	1,05E-02
Tbcel	up	1,06	6,01 E-01	up	1,10	4,47E-01	down	1,09	6,51E-01
Nardo et al. 2013	Presymptomatic 129sv G93A vs. Ctrl			Onset 129sv G93A vs. Ctrl			Symptomatic 129sv G93A vs. Ctrl		
Gene	Regulation	Fold-Change	*P*-Value	Regulation	Fold-Change	*P*-Value	Regulation	Fold-Change	*P*-Value
Tbca	up	1,11	5,19E-01	down	1,11	2,51 E-01	down	1,10	4,67E-01
Tbcb	up	1,22	2,89E-01	down	1,45	3,00E-03	down	1,45	1,61E-02
Tbcc	up	1,20	1,50E-01	down	1,23	4,28E-02	down	1,06	3,85E-01
Tbcd	up	1,28	1,37E-01	down	1,11	4,26E-01	down	1,06	5,97E-01
Tbce	up	1,17	2,44E-01	down	1,20	8,76E-03	down	1,05	5,69E-01
Tbcel	up	1,10	6,18E-01	up	1,01	9,11E-01	down	1,02	8,61E-01

Gene expression profiles from mutant SOD1 G93A and control motor neurons isolated by laser capture microdissection at different stage of disease [19, 40] respectively were downloaded from the Gene Omnibus database and analyzed with EASANA software based on FAST DB annotations [12, 13]. Gene dysregulation was considered significant at fold changes >1.5 and *p*-values < 5E-02

Genes encoding tubulin-binding cofactors Tbca to Tbcel are not significantly dys-regulated in mutant SOD1 motor neurons at any time point in the datasets GSE10953 [19] or GSE46298 [40]

Note that mutant SOD1 G93A mice with genetic backgrounds C57BL/6 or 129sv and corresponding control mice were analyzed in the study of Nardo and colleagues [40]. Here, onset of disease was defined when mice showed first signs of paw grip strength impairment and when body weight started to decline. Symptomatic disease was defined when mice displayed a decrease of 50 % in their latency on grip strength and when body weight declined by >5 %

By contrast, we found three Stathmin genes whose expression was significantly modulated in presymptomatic mutant SOD1^G93A^ motor neurons when compared to control. Stathmin 1 and Stathmin 2 were up-regulated by 1.87 and 1.63 fold respectively, whereas Stathmin 3 was down-regulated by 1.71 fold at P60 (p < 0.05, Table 2). Stathmin 1 up-regulation (by 1.68 fold) also tended to occur at end stage in an independent dataset of Perrin et al. [42], although not reaching statistical significance (p = 0.06, Table 2).

**Table 2:** Gene expression profiles of Stathmins in mutant SOD1 motor neurons

Ferraiuolo et al. 2007	Presymptomatic G93A vs. Ctrl (P60)			Symptomatic G93A vs. Ctrl (P90)			Endstage G93A vs. Ctrl (P120)		
Gene	Regulation	Fold-Change	*P*-Value	Regulation	Fold-Change	*P*-Value	Regulation	Fold-Change	*P*-Value
Stmnl	**up**	**1,87**	**3,80E-02**	up	1,21	4,54E-01	down	1,40	3,32E-01
Stmn2	down	1,03	9,47E-01	down	1,39	2,65E-01	up	1,91	3,96E-01
Stmn3	**down**	**1,71**	**9,50E-03**	down	1,44	2,73E-01	down	1,21	2,92E-01
Stmn4	down	1,02	9,61 E-01	up	1,39	5,15E-01	up	1,09	8,50E-01
Nardo et al. 2013	Presymptomatic C57 G93A vs. Ctrl			Onset C57 G93A vs. Ctrl			Symptomatic C57 G93A vs. Ctrl		
Gene	Regulation	Fold-Change	*P*-Value	Regulation	Fold-Change	*P*-Value	Regulation	Fold-Change	*P*-Value
Stmnl	down	1,35	9,31 E-02	up	1,17	1,49E-01	up	1,16	3,48E-01
Stmn2	down	1,05	8.98E-01	down	1,52	2,38E-01	up	1,36	4,89E-01
Stmn3	up	1,07	7.28E-01	down	1,24	1,41 E-02	down	1,14	1,50E-02
Stmn4	down	1,06	6,69E-01	up	1,43	1,63E-01	up	1,36	3,15E-02
Nardo et al. 2013	Presymptomatic 129v G93A vs. Ctr			Onset 129sv G93A vs. Ctrl			Symptomatic 129sv G93A vs. Ctrl		
Gene	Regulation	Fold-Change	*P*-Value	Regulation	Fold-Change	*P*-Value	Regulation	Fold-Change	*P*-Value
Stmnl	up	1,05	5,50E-01	up	1,06	3,40E-01	down	1,04	7,57E-01
Stmn2	**up**	**1,63**	**1.98E-02**	down	1,46	1,76E-01	down	1,71	9,08E-02
Stmn3	up	1,08	6,24E-01	down	1,25	6,38E-03	down	1,30	1,18E-02
Stmn4	down	1,13	3,75E-01	up	1,15	3,11E-01	up	1,23	2,98E-03
Perrin et al. 2006	Presymptomatic G93A vs. Ctrl (P60)			Symptomatic G93A vs. Ctrl (P90)			Endstage G93A vs. Ctrl (P120)		
Gene	Regulation	Fold-Change	*P*-Value	Regulation	Fold-Change	*P*-Value	Regulation	Fold-Change	*P*-Value
Stmnl	down	1,38	nc	down	1,38	nc	up	1,68	6,00E-02
Stmn2	up	1,20	nc	up	1,30	1,36E-01	up	1,12	6,44E-01
Stmn3	down	1,03	nc	up	1,07	5,63E-01	down	1,02	8,36E-01
Stmn4	up	1,75	nc	up	1,30	6,49E-01	up	1,85	nc

Gene expression profiles from mutant SOD1 and control motor neurons were analyzed with EASANA software based on FAST DB® annotations, see Table 1. Gene dysregulation was considered significant at fold changes >1.5 and *p*-values < 5E-02. nc: non calculated

The Stmn1 and Stmn2 genes are significantly up-regulated at presymptomatic disease stage in datasets GSE10953 [19] and GSE46298/129sv [40] respectively, whereas the Stmn3 gene is significantly down-regulated in dataset GSE10953 [19]. Stathmin-1 also tended to be up-regulated in the dataset kindly provided by F. Perrin [42]

Stathmins represent a family of four proteins (Stathmins 1 to 4), which destabilize microtubules either by sequestering tubulin dimers or by increasing the frequency of microtubule catastrophes, i.e. their transition from steady growth to rapid depolymerization [3, 10]. All Stathmins are highly expressed in neurons, several of them localize to the Golgi [10] and Stathmin 1 protein levels were found increased in spinal cords of paralyzed (late stage) SOD1^G93A^ mice [55]. We therefore considered Stathmins as good candidates for mutant SOD1-linked microtubule alterations and Golgi fragmentation.

**Fig. 4:**
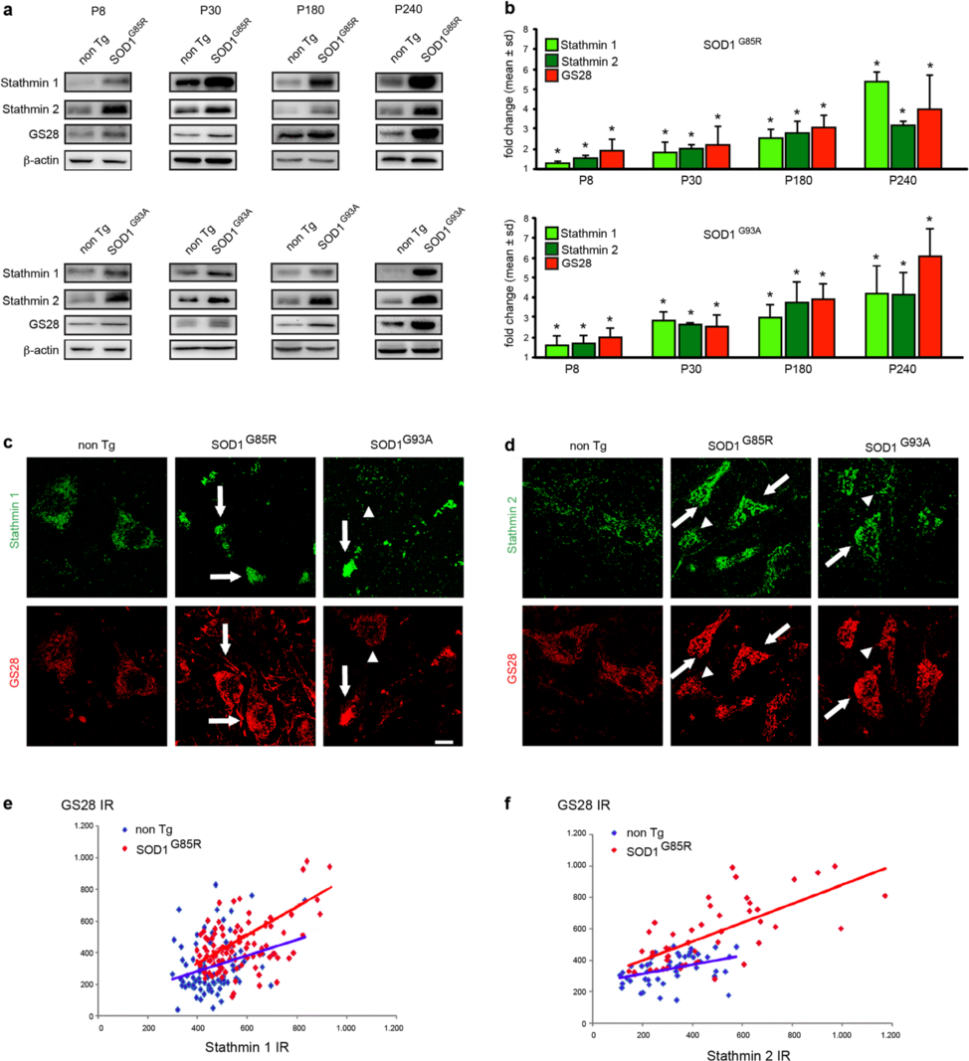
Early and rapidly progressive co-accumulation of Stathmin 1, Stathmin 2 and GS28 in motor neurons of mutant SOD1 mice. **a**. Western blots show expression of Stathmin 1, Stathmin 2 and GS28 in lumbar spinal cords of SOD1^G85R^ mice (upper panels), SOD1^G93A^ mice (lower panels) and corresponding litter mates. Analyses were performed at ages P8, P30, P180 and P240. Loading control β-actin. Each blot was performed in duplicate and is representative of four animals per genotype. **b**. Diagrams showing kinetics of Stathmin 1, Stathmin 2 and GS28 protein levels in lumbar spinal cords of mutant SOD1^G85R^ mice and SOD1^G93A^ mice. Stathmin 1 and 2 levels are already significantly increased at P8. Fold changes (mean ± sd) are determined from four spinal cords per genotype and time point and expressed relative to the levels in non-transgenic littermate controls (set to 1). Differences between mutant SOD1 and control are statistically significant as measured by Mann Whitney test (*, p < 0.01). **c**-**d**. Confocal images of lumbar spinal cord cross sections from non-transgenic, SOD1^G85R^ and SOD1^G93A^ mice aged 240 days showing accumulation of Stathmin 1 (C, upper panels), Stathmin 2 (D, upper panels) and GS28 (C-D, lower panels) in motor neurons, which sometimes appear as degenerating. Note motor neurons with either low expression (arrowheads) or high expression (arrows) of Stathmins and Golgi SNAREs. Scale bar 20 μm. **e**. Diagram showing immunoreactivities (IR) of Stathmin 1 (x-axis) and GS28 (y-axis) in motor neurons of control non-transgenic mice (in blue) and mutant SOD1^G85R^ mice (in red). Pearson analysis demonstrates significant correlation between both parameters: r = 0.28, p < 0.0066 (ctrl) and r = 0.47, p < 0.0001 (SOD1^G85R^). The slopes of the regression curves are 0.49 ± 0.18 (ctrl) and 0.68 ± 0.13 (SOD1^G85R^), n = 91 cells (ctrl) and n = 99 cells (SOD1^G85R^) analyzed from n = 3 mice per genotype. **f**. Immunoreactivities of Stathmin 2 and GS28 also show significant correlation by Pearson analysis: r = 0.40, p < 0.0047 (ctrl), r = 0.69, p < 0.0001 (SOD1^G85R^). The slopes of the regression curves are 0.29 ± 0.09 (ctrl) and 0.60 ± 0.09 (SOD1^G85R^) by linear regression analysis, n = 47 cells and n = 48 cells analyzed respectively from n = 3 mice per genotype

We analyzed whether one of the four Stathmins is dysregulated at the protein level in the spinal cord of mutant SOD1 mice. Using Western blot, we found that levels of Stathmin 1 (19 kD) and Stathmin 2 (also called SCG10, ~22 kD) are both increased by more than 3 fold in mutant SOD1^G85R^ and SOD1^G93A^ spinal cords at age 240 days (Fig. 4a) whereas they are not in SOD1^wt^ mice (not shown). Of note, this up-regulation is not observed for Stathmin 3 (RB3, ~26 kD) (not shown) and Stathmin 4 (RB3’, ~24 kD) is undetectable. We therefore validate that expression of mutant SOD1 triggers up-regulation of Stathmins 1 and 2 in motor neurons and conclude that this could cause the observed microtubule defects and Golgi fragmentation.

### Early and progressive co-accumulation of Stathmins and GS28 in mutant SOD1 motor neurons

We then set out to test whether Stathmin up-regulation could be causative of the Golgi fragmentation. As a first prerequisite to this, Stathmin up-regulation should have kinetics matching the early onset and progression of Golgi pathology in mutant SOD1 spinal cord [36, 65, 66]. To establish this, we monitored the kinetics of Stathmin 1 and Stathmin 2 protein levels from early pre-symptomatic stage (postnatal days P8 and P30) to disease endstage (P240) and compared it to GS28 increase as a molecular sign of Golgi pathology. The levels of Stathmin 1 and 2 in spinal cords of mutant SOD1^G85R^ and SOD1^G93A^ mice are already significantly increased at P8 when compared to non-transgenic litter mice (Fig. 4a-b). Their levels continue to rise at P30 and P180 when compared to non-transgenic mice to finally reach >3 fold normal levels at P240 (Fig. 4a-b).

Remarkably, this completely parallels the increase in GS28 protein level with a 1.5 fold increase at P8 and a 4 to 6 fold increase at P240 (Fig. 4a-b). The accumulation of Stathmins 1/2 and GS28 thus precedes by at least five months the first clinical symptoms in two different lines of mutant SOD1 mice. These data confirm that Golgi pathology is an early preclinical sign in mutant SOD1 mice and show that the microtubule-disrupting proteins Stathmin 1 and 2 are progressively up-regulated at kinetics compatible with Golgi fragmentation.

As a second prerequisite, Stathmin up-regulation should specifically occur in motor neurons displaying Golgi SNARE accumulation. To test this, we identified individual motor neurons by choline acetyl transferase (ChAT) expression (Additional file 1: Figure S4A-B) and examined the levels of Stathmins and GS28 by IF on a cell-to-cell basis (Fig. 4c-d). In normal motor neurons, Stathmin 1 is barely detectable, Stathmin 2 is expressed in a faint punctate fashion (Fig. 4c-d, upper left panels) and GS28 levels are also low. In contrast, the increase in Stathmin 1 or 2 labeling intensities seen in numerous motor neurons of mutant SOD1 mice (Fig. 4c-d, upper middle and right panels, arrows) is matched by an increase in GS28 (Fig. 4c-d, lower middle and right panels, arrows, Additional file 1: Figure S4A-B). Interestingly, no up-regulation of GS28 or Stathmins 1/2 was apparent in neighboring ChAT-negative interneurons (Additional file 1: Figure S4A-B).

We also used regression analysis to demonstrate a clear correlation between the immunoreactivities of Stathmin 1 and GS28 (Fig. 4e) and between Stathmin 2 and GS28 (Fig. 4f) in individual mutant SOD1 motor neurons.

As a third prerequisite, Stathmin 1 and 2 should be expressed where microtubules are nucleated or polymerized. In line with earlier reports [9], we find that Stathmin 2 is localized to the MannII-GFP-labelled Golgi in NSC-34 motor neurons whereas Stathmin 1 is mostly cytosolic (Additional file 1: Figure S5).

In summary, the kinetics of Stathmin 1/2 up-regulation and its close temporal and spatial correlation with GS28 accumulation in mutant SOD1 motor neurons all suggest that Stathmin up-regulation may mediate Golgi fragmentation.

### Overexpression of Stathmins 1/2 phenocopies expression of mutant SOD1

**Fig. 5:**
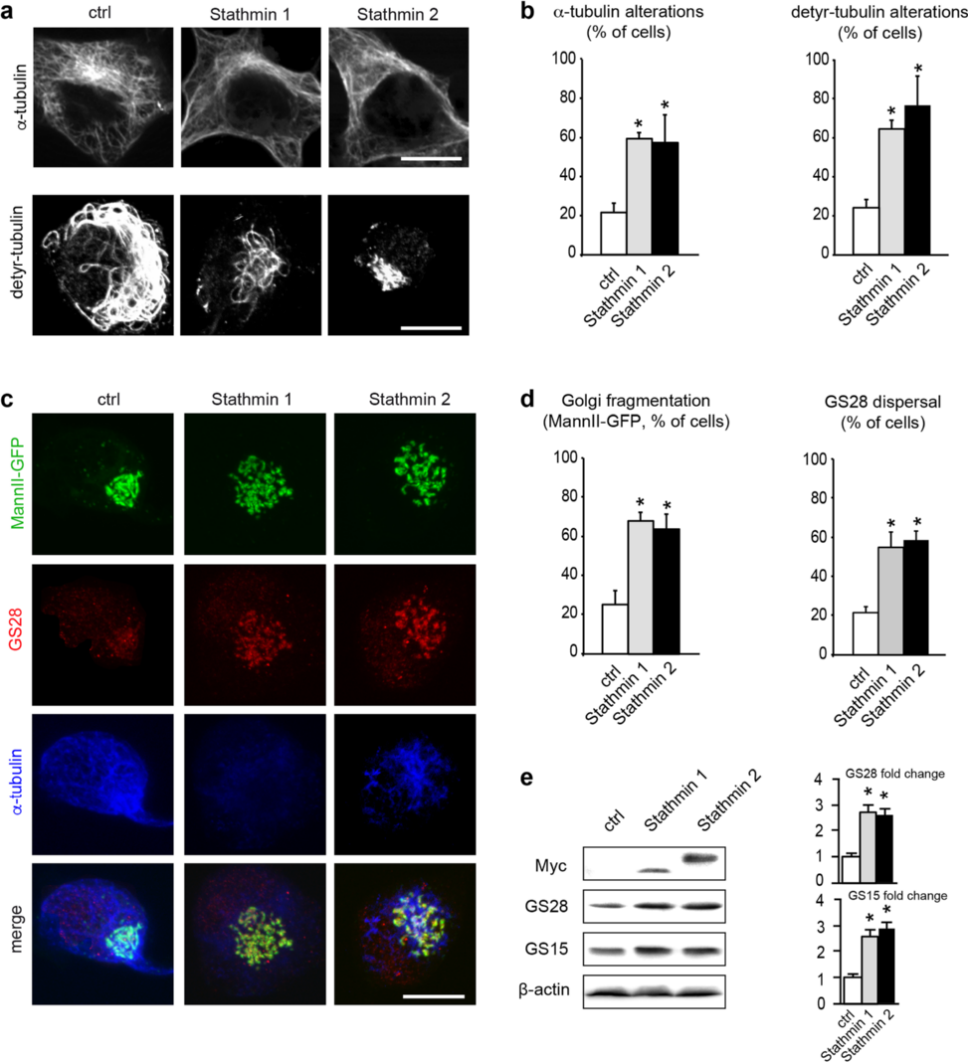
Stathmin 1/2 overexpression phenocopies expression of mutant SOD1. **a**. Images showing microtubule alterations in NSC-34 motor neurons transfected with Myc-tagged Stathmin 1 or Stathmin 2, as compared to control cells. Cells were cultured for 2 DIV, fixed with paraformaldehyde and analyzed with antibodies α-tubulin (upper panels), detyrosinated tubulin (lower panels) or Myc (not shown). Scale bar 5 μm. **b**. Quantitative analyses showing percentage of cells with rarefied or broken microtubules (α-tubulin or detyrosinated tubulin). Data represent one typical out of five independent experiments each done in quadruplicate per condition. Number of cells analyzed (ctrl, Stathmin 1, Stathmin 2): α-tubulin (175, 174, 139), detyr-tubulin (184, 146, 152). Statistical significance by Mann–Whitney test: * p < 0.001 as compared to mock. **c**. Images showing Golgi alterations (MannII-GFP, GS28) together with microtubule alterations (α-tubulin) in NSC-34 motor neurons transfected with Stathmin 1 or Stathmin 2 as compared to control cells. Scale bar 5 μm. **d**. Quantitative analyses showing percentage of cells with Golgi alterations (MannII-GFP) or GS28 dispersal). Data represent one typical out of five independent experiments each done in quadruplicate per condition. Number of cells analyzed (ctrl, Stathmin 1, Stathmin 2): MannII-GFP (>500 each). GS28 (482, 434, 526). Statistical significance by Mann–Whitney test: * p < 0.001 as compared to mock. **e**. Western blot analysis of cells transfected with plasmids encoding Myc-tagged Stathmin 1 or Stathmin 2 or with an empty control plasmid (pcDNA). GS15 and GS28 levels are increased after overexpression of Stathmin 1 by 2.7 ± 0.3 and 2.6 ± 0.3 (mean ± sd) respectively. A similar increase in Golgi SNAREs is observed after overexpression of Stathmin 2 (GS15 2.6 ± 0.2, GS28 2.9 ± 0.3). n = 4 blots corresponding to independent experiments, * p < 0.01 by Mann - Whitney test

If Stathmin 1/2 up-regulation is responsible for the Golgi fragmentation observed in mutant SOD1 motor neurons through its effect on the microtubule network, it should lead on its own to the structural and molecular alterations described above, i.e. microtubule disruption, Golgi fragmentation and Golgi SNARE accumulation. We first confirmed that Stathmin 1/2 overexpression in NSC-34 motor neurons leads to the expected microtubule defects. Indeed, we found that the percentage of Stathmin 1 or 2 overexpressing NSC-34 cells with altered microtubules is 3 fold higher than in control-transfected cells (Fig. 5a-b). This is true for microtubules containing α-tubulin or detyrosinated tubulin and similar to that in mutant SOD1-transfected cells (Fig. 3a,b,c,f).

We then tested whether Stathmin 1/2 overexpression in NSC-34 cells triggers Golgi fragmentation and the associated accumulation of Golgi SNAREs GS28 and GS15 (Fig. 1g-i and Fig. 4). We found that it is the case. About 60 % of Stathmin 1 transfected cells exhibit a fragmented Golgi and GS28 dispersal (Fig. 5c-d) and the protein levels of GS28 and GS15 are >2.5 fold higher than in control cells (Fig. 5e). Stathmin 2 transfected cultures show a similar percentage of cells with Golgi fragmentation (Fig. 5c-d) and a similar increase in GS28/GS15 protein levels (Fig. 5e). Taken together, these data show that Stathmin 1 and Stathmin 2 overexpression on its own triggers both microtubule and Golgi alterations that qualitatively and quantitatively resemble those observed in mutant SOD1 motor neurons.

### Knockdown of Stathmins 1 or 2 rescues mutant SOD1-linked Golgi fragmentation

To conclusively validate the role of Stathmin 1/2 up-regulation in mutant SOD1-triggered Golgi fragmentation, we reasoned that removing Stathmins in mutant SOD1-expressing NSC-34 cells should prevent microtubule loss and Golgi fragmentation.

**Fig. 6:**
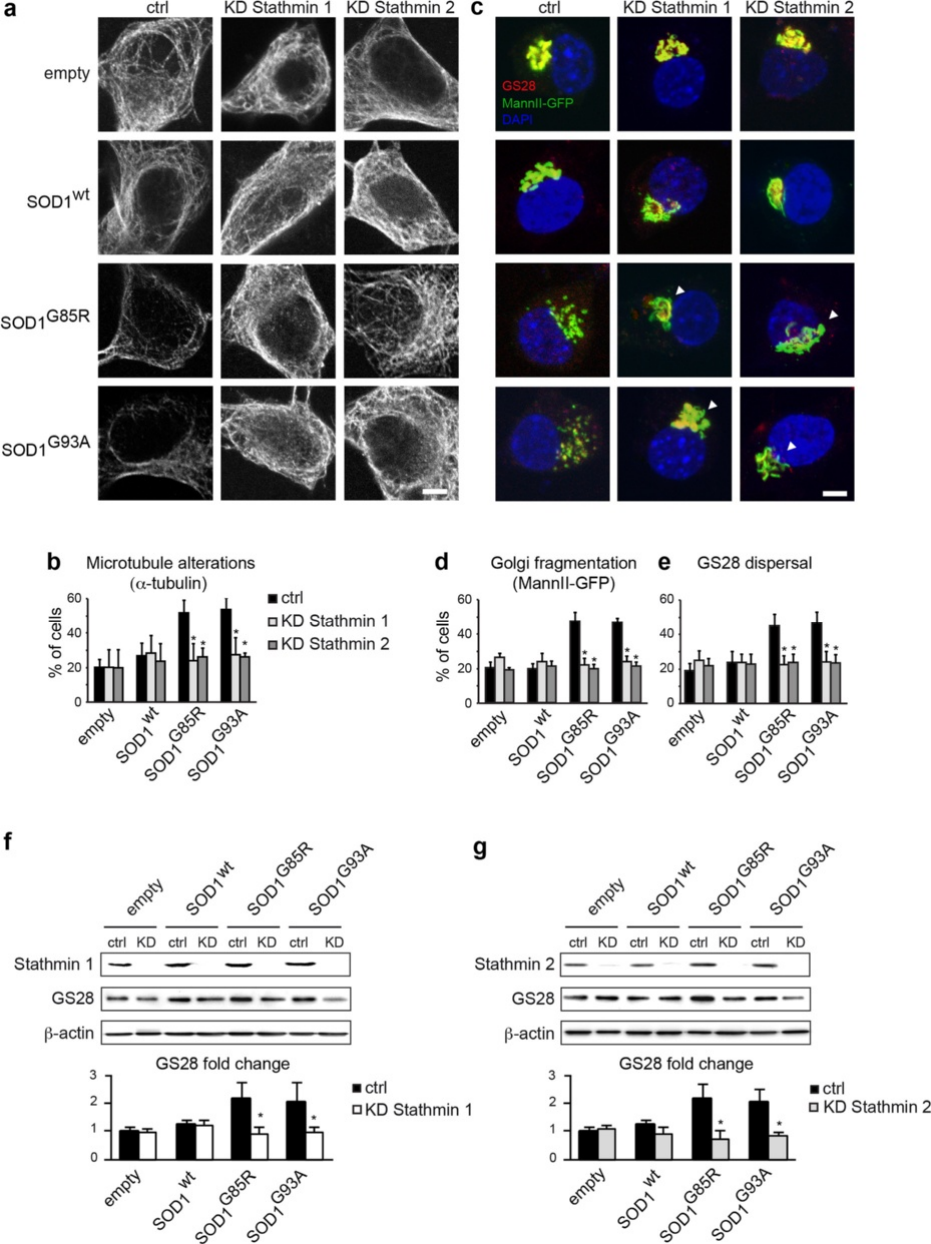
Stathmin 1/2 knockdown rescues microtubule and Golgi alterations triggered by mutant SOD1. **a**. Confocal images showing NSC-34 cells transfected with empty, SOD1^wt^, SOD1^G85R^ or SOD1^G93A^ plasmids as well as ctrl, Stathmin-1 or Stathmin-2 siRNAs. Cells were immunolabelled for α-tubulin. Note that knockdown of Stathmin 1 or 2 rescues mutant SOD1-triggered microtubule alterations. Transfected cells were identified by expression of co-transfected MannII-GFP (not shown). Scale bar 5 μm. **b**. Diagram showing percentage of microtubule alterations (mean ± sd) under the different conditions. >150 cells were analyzed per condition. Statistical significance * p < 0.01 by Mann–Whitney test. Data are representative of three independent experiments. **c**. Confocal images showing NSC-34 cells transfected as in A and counterstained for GS28. Note rescue (arrowheads) of mutant SOD1-triggered Golgi fragmentation and GS28 dispersal by knockdown of Stathmin 1 or 2. Scale bar 5 μm. **d**-**e**. Diagram showing percentage (mean ± sd) of Golgi alterations labelled by MannII-GFP (**d**) or GS28 (**e**) under the different conditions. >150 cells were analyzed per condition. Statistical significance * p < 0.01 by Mann–Whitney test. Data are representative of four independent experiments. **f**-**g**. Western blots show efficient siRNA-mediated knockdown (KD) of Stathmin 1 (**f**) and Stathmin 2 (**g**) in NSC-34 cells transfected with empty, SOD1^wt^, SOD1^G85R^ or SOD1^G93A^ plasmids as compared to ctrl siRNA (upper blots). Both Stathmin-1 or Stathmin-2 knockdown reduces the GS28 up-regulation triggered by SOD1^G85R^ or SOD1^G93A^ (middle blots). Loading control β-actin (lower blots) indicates reduced protein content in lanes 1 and 2 in F (empty ctrl, empty Stathmin-1 KD). The diagrams below show densitometric quantification of GS28 protein levels each normalized to β-actin (n = 4 blots, mean ± sd, * statistically significant differences between ctrl siRNA and Stathmin 1/2 siRNA as measured by Mann - Whitney test)

We found that RNAi-mediated knockdown of either Stathmin 1 or Stathmin 2 reduces the percentage of cells with mutant SOD1-triggered microtubule alterations such as rarefied or discontinous microtubules to control values (Fig. 6a-b). We further show that the knockdown of either Stathmin 1 or 2 rescues the morphological Golgi fragmentation evidenced by MannII-GFP (Fig. 6c) and also the vesicular dispersion of GS28-labeled Golgi profiles (Fig. 6c). Quantitative analyses (Fig. 6d-e) demonstrate a complete rescue of both types of alterations.

Finally, we demonstrate that Stathmin 1 knockdown normalizes the pathologically increased levels of GS28 triggered by SOD1^G85R^ or SOD1^G93A ^to those in SOD1^wt^ cultures (Fig. 6f). Stathmin 2 knockdown has a similar effect (Fig. 6g). Taken together, these data indicate that Stathmins 1/2 mediate Golgi alterations in mutant SOD1 motor neurons by triggering microtubule loss.

## Discussion

Our study shows that the early Golgi pathology observed in mutant SOD1 motor neurons is triggered by the up-regulation of Stathmins 1/2 that sever the Golgi-nucleated microtubule network. This in turn leads to Golgi fragmentation with tubular/vesicular organelle transformation as its most prominent ultrastructural feature and strong accumulation of Golgi SNAREs GS28 and GS15 as its most striking molecular signature.

### Mutant SOD1-linked Golgi fragmentation is due to “somatic microtubulinopathy”

Surprisingly, we find that Golgi fragmentation in mutant SOD1 motor neurons *in vivo* and *in vitro* resembles that described in *pmn* mice mutated in the Golgi-localized tubulin folding chaperone TBCE, leading to TBCE degradation and defective polymerization of Golgi-derived microtubules in the cell soma [5]. This was unexpected since microtubule alterations in mutant SOD1-linked ALS have so far been only reported in axons, as illustrated by a reduction in axonal tubulin transport [70], an accumulation of β_III_-tubulin in axonal swellings [70] and increases in hyperdynamic microtubules [17] or density of EB3 comets [27] in axons. By contrast, our findings show that mutant SOD1 impedes microtubule dynamics at the Golgi in the motor neuron soma, leading in turn to Golgi fragmentation. Of note, some forms of human ALS are associated with mutations in the α-tubulin gene TUBA4A and these severely disrupt the somatic microtubule network upon overexpression [51]. We therefore propose *“somatic microtubulinopathy“ *as a common mechanism triggering Golgi fragmentation in SOD1-linked ALS, progressive motor neuronopathy, human TUBA4A-linked ALS, and similar disorders due to mutations yet to be identified.

### Defective microtubules in mutant SOD1 motor neurons via stathmin 1 and 2 up-regulation

How does mutant SOD1 induce somatic microtubulinopathy? We show that mutant SOD1 disrupts the cellular microtubule network through up-regulation of the microtubule-disrupting proteins Stathmin 1 and 2. This up-regulation is specific to mutant SOD1 since wildtype SOD1 has no such effect, and it is independent of SOD1 enzymatic activity since dismutase-inactive SOD1^G85R^ has a similar effect as dismutase active SOD1^G93A^.

Interestingly, wildtype and mutant forms of SOD1 have been localized to the Golgi by biochemical and imaging methods [63, 64]. We hypothesize that Golgi-localized mutant SOD1 triggers a Golgi stress response to the nucleus, in analogy to the Golgi-disrupting agents Brefeldin A and monensin, which activate the signaling cascades CREB3/ARF4 [45] and TFE3/GASE [59], respectively. According to our bioinformatic analyses, mutant SOD1 does not modulate the gene expression of the transcription factors CREB3 and TFE3 or their respective effectors ARF4 and GCP60 (ACBD3), (data not shown). However, mutant SOD1 enhances gene expression of EZH2 (enhancer of zeste homolog 2), a known de-repressor of the Stathmin 1 gene [11], by 1.65 fold and 1.56 fold in motor neurons of two transgenic lines (p = 0.014 and p = 0.003, dataset GSE46298 [40], providing a potential mechanism for Stathmin 1 up-regulation. In addition, mutant SOD1 may influence the Stathmin 1 activity through posttranslational modification of its isoforms [55]. These findings point to a novel Golgi stress response pathway in ALS that needs to be characterized in further detail.

### The respective contribution of Stathmins 1 and 2 to Golgi fragmentation

Our data show that knockdown of Stathmin 1 or 2 completely rescues molecular and morphological Golgi alterations. This rules out that Stathmin 1/2 up-regulation is merely a homeostatic mechanism during Golgi fragmentation, similar to Stathmin up-regulation after axonal injury or during axonal regeneration [25, 32, 49].

We further demonstrate that Stathmin 1 or 2 overexpression has similar effects on Golgi fragmentation and Golgi SNARE accumulation in motor neurons. Yet, Stathmins 1 and 2 differ in their subcellular localization in motor neurons (Additional file 1: Figure S4) and in other cell types [10]. While Stathmin 1 appears mainly cytoplasmic, Stathmin 2 localizes to the Golgi due to its N-terminal domain which is missing in Stathmin 1 [9]. This suggests that these two Stathmins fulfill different functions and potentially act on distinct microtubule subsets. According to recent studies, radial centrosomal microtubules are required for the proper positioning of the Golgi in the cell center whereas microtubules nucleated at the trans-Golgi by CLASPs [14, 34] or at the cis-Golgi by AKAP450/GM130 [46] seem to be required for the lateral continuity of Golgi membranes. It can be speculated that up-regulated Stathmins 1 and 2 cooperatively trigger Golgi fragmentation by regulating interdependent microtubule subsets required for Golgi maintenance.

### Are there additional mediators of mutant SOD1-linked Golgi fragmentation?

Our data indicate that Golgi fragmentation in mutant SOD1 motor neurons is associated with displacement of GM130 from intact Golgi membranes to the cytosol (Fig. 1g). A recent study in mitotic cells shows that GM130 stimulates microtubule polymerization by activating the microtubule nucleator TPX2 through sequestration of its inhibitor importin α [67]. Displacement of GM130 to the cytosol with ensuing TPX2/importin α imbalance may thus contribute to mutant SOD1-linked microtubule loss and Golgi fragmentation. Furthermore, mutant SOD1 has been reported to interact aberrantly with chromogranins [64] and the COPII vesicle subunit Sec23 [4], which may impede ER-Golgi and Golgi-plasma membrane transport [4, 54, 64] and further enhance Golgi fragmentation.

### Stathmins as potential biomarkers for ALS

Last, this study may contribute to the development of new disease biomarkers. Biomarkers are generally defined as a physiological process, a pathogenic event or a pharmacological response to therapeutic intervention that can be measured. Reliable biomarkers for ALS are currently lacking [41, 62]. In this respect, Stathmins display several interesting features.

First, levels of Stathmins 1 and 2 are already significantly elevated at an early preclinical phase in two lines of mutant SOD1 mice - five months before appearance of clinical symptoms (Fig. 4a-b). Translating this to human ALS patients would help to establish earlier diagnosis and initiate therapies at a more tractable disease phase.

Second, both Stathmins strongly and continuously accumulate during disease course in mutant SOD1 G85R and G93A spinal cords, reaching levels of > 3 fold above normal at endstage (this study, Fig. 4a-b). To our knowledge, this is higher than for any previously reported protein in ALS spinal cord, and may allow sensitive monitoring of disease progression.

Third, Stathmin accumulation represents a pathologically relevant feature shared between SOD1-linked ALS and mutant SMN-linked spinal muscular atrophy (SMA) [68]. Genetic knockout of Stathmin 1 in *Smn* mice improved neuromuscular function presumably through microtubule stabilization [69], underscoring the pathogenic relevance of Stathmin 1 up-regulation.

Finally, the proteins Stathmin 1 and 2 are both present in normal human blood (Plasma Proteome Database [39]) with Stathmin 1 being measured at a plasma concentration of 3.5 ng/ml [18]. Pathological release of Stathmins from degenerating ALS motor neurons may thus cause a detectable increase in their blood levels.

Stathmins and dys-regulated Golgi proteins should therefore be evaluated as new potential biomarkers for diagnosis, prognosis or therapy response in ALS, SMA and related motor neuron disorders.

## Conclusions

This study demonstrates that Stathmin 1/2-triggered microtubule destabilization mediates early presymptomatic Golgi fragmentation in mutant SOD1 G85R- and G93A-triggered ALS. This may contribute to the development of new blood biomarkers in ALS and related motor neuron diseases.

## Methods

### Antibodies and reagents

Primary and secondary antibodies are listed in Additional file 1: Table S1. Other reagents were from the following suppliers: PBS, Hbss, trypsin, culture media and supplements (Invitrogen, Carlsbad, CA), taxol, nocodazole (Sigma), OCT (Thermo Scientific, Runcorn, UK), Vectashield (Vector laboratories, Burlingame, CA), Complete protease inhibitors (Roche, Basel, Switzerland), Ketamine (Bayer, Leverkusen, Germany) and Xylazine (Mérial, Lyon, France), coverslips and Superfrost Plus glass slides (Menzel, Schwerte, Germany). Plasmid expression vectors contained the following cDNAs: GFP-tagged Mannosidase-II (Dr. M. Bornens, Institut Curie, Paris, France), untagged human SOD1^wt^, SOD1^G85R^ or SOD1^G93A^ (Dr. D. Borchelt, University of Florida, Gainesville, USA) subcloned in pCAGGS vectors, RFP-tagged human SOD1^wt^, SOD1^G85R^ or SOD1^G93A^ (Dr. J. Weishaupt, Neurologische Klinik, Ulm, Germany), Myc-tagged human Stathmin 1 or Stathmin 2 (Dr. A. Sobel, Inserm U693, Paris, France).

### Animals

Transgenic SOD1^G93A^ mice (line G1del or Tg(SOD1*^G93Adl^)1Gur, http://jaxmice.jax.org/strain/002299.html [1]), SOD1^G85R^ mice (line 148 [6] and SOD1^wt^ mice (line 76 [6]) were maintained as hemizygotes for more than ten generations on a C57/BL6 background and genotyped by PCR as described [44]. All experiments with animals were performed in strict compliance with French and European legislation.

### Cell culture

NSC-34 cells [7] were cultured in DMEM supplemented with 8 % (v/v) fetal calf serum at 37 °C and 7.5 % CO_2_and differentiated on collagen-coated wells in low fetal calf serum (1 %) for 72 h before transfection. Cells were transfected with plasmids encoding SOD1 (mutant or wildtype, RFP-tagged or not), RFP, Stathmin-1 or Stathmin-2 (0.2 μg/10^4^ cells) and/or with siRNAs against Stathmin 1/2 (on target plus, Thermofisher) or luciferase control (Thermofisher) using Lipofectamine 2000 (Invitrogen) as described [48]. Cells were then cultured for 96 h after SOD1 plasmid and/or Stathmin siRNA transfection or for 48 h after Stathmin plasmid transfection.

### Immunocytochemistry

Mice deeply anaesthetized with ketamine/xylazine were intracardially perfused with Sorensen buffer followed by 4 % paraformaldehyde. Spinal cords were dissected out, postfixed, cryoprotected in sucrose and frozen in OCT. Cross sections (30 μm) of lumbar spinal cord were cut with a crytotome (Leica) and stained with antibodies as described [5].

Cultured cells were fixed in 4 % formaldehyde (FA), permeabilized with 0.5 % (v/v) Tween in PBS and blocked in a solution containing 2.5 % (v/v) goat serum, 2.5 % (v/v) donkey serum, 1 % (w/v) BSA in PBS. Alternatively, cells were extracted for soluble proteins using MSB (25 mM Hepes, 50 mM Pipes, 10 mM EGTA, 2 mM MgCl_2_, 1 % formaldehyde in PBS pH 7.0), permeabilized in MSB containing 10 μM Taxol and 0.2 % (v/v) Triton X100 and fixed with 4 % formaldehyde. Cells were stained with antibodies as described [5].

### Confocal imaging, morphometry and 3D modeling

Motor neurons in ventral spinal cord were identified by expression of vesicular acetyltransferase (VAChT) or choline acetyltransferase (ChAT), large soma size and faintly DAPI-stained large nucleus [5]. Motor neurons were imaged with an LSM 510 confocal microscope (Zeiss, Oberkochen, Germany) using a 63x objective, an xy-resolution of 1024 x 1024 pixel and a z-interval of 0.3 μm.

 Morphometric analyses were done with Metamorph software (Molecular Dynamics) using single confocal cross sections taken at the nuclear midplane. The boundaries of the VAChT-labelled cell soma and the DAPI-stained nucleus were manually delineated with the Metamorph drawing tool. Golgi area was automatically determined with the Metamorph morphometry tool by applying a fixed threshold to the GM130 signal. Three-dimensional (3D) modelings of the Golgi apparatus were done with Imaris software (Bitplane, Zurich, Switzerland). Images were processed and Golgi membranes visualized using the IsoSurface mode of the Surpass module. The number of individual Golgi elements per motor neuron was determined in a blinded manner. NSC-34 cells were imaged by confocal microscopy using a z-interval of 0.3 μm and a scan depth of about 10 μm.

The percentage of spinal cord motor neurons displaying Golgi fragmentation (labelled by GM130 or MG160) was determined by conventional microscopy using a Leica DMI 400 fluorescence microscope (63x oil objective). Golgi fragmentation in lumbar spinal cord motor neurons was defined by discontinuous or decreased GM130 or MG160 immunolabeling.

The percentage of transfected NSC-34 cells with subcellular alterations was also quantified by fluorescence microscopy. Golgi dispersal was defined by enlarged area, as compared to neighboring non-transfected cells. A defective microtubule network was defined by less dense or discontinuous microtubules labeled for α-tubulin or detyrosinated tubulin, as compared to neighboring non-transfected cells.

### Electron microscopy

Deeply anaesthetized mice were transcardially perfused with Sorensen’s phosphate buffer (pH 7.4) followed by glutaraldehyde (2 % v/v in cacodylate). Spinal cords were dissected out, postfixed for 24 h and cut into small segments comprising the ventral spinal cord before processing for resin embedding (epon) following standard protocols. 60 nm ultrathin cross sections were contrasted with uranyl acetate and visualized under a JEOL electron microscope. Two sections each containing up to 30 motor neurons were analyzed per mouse line. Motor neurons were recognized on the basis of their frequency (1:20), large size and pale nucleus with nucleolus.

### Immunoblots and subcellular fractionation

Protein extracts from lumbar spinal cords or NSC-34 cells were prepared by homogenization in lysis buffer containing 50 mM Tris HCl pH7.5, 150 mM NaCl, 2 mM EDTA, 1 % Triton X100, protease inhibitors (Complete EDTA-free, Roche). 50 μg protein were subjected to SDS-PAGE and blotted on Immobilon membranes (Millipore) which were processed by standard methods and revealed with Immobilon Western kits (Millipore). Band intensities were quantified by TotalLabQuant software.

Crude fractionation of membranes from lumbar spinal cord was performed after tissue freezing (−80 °C), thawing and homogenization in 50 mM HEPES, pH 7.4, 250 mM sucrose, 1 mM Mg-acetate and protease inhibitors (Complete EDTA-free, Roche). Lysates were homogenized using a Dounce homogenizer (15 passes) and centrifuged at 1.000 g for 10 min. The postnuclear supernatant was centrifuged at 10.000 g for 30 min at 4 °C yielding a P10 pellet and the supernatant was centrifuged at 100.000 g (Beckman TLA-110) for 1 h at 4 °C yielding an S100 supernatant and a P100 pellet.

### Tubulin and microtubule assays

Tubulin polymerization status in NSC-34 cells was analyzed by preparing cell lysates in microtubule stabilization buffer (MSB, 0.1 M PIPES pH 6.75, 1 mM EGTA, 1 mM MgSO_4_, 30 % glycerol, 5 mM GTP, 5 % DMSO, 1 mM DTT, MiniComplete protease inhibitors). Total tubulin (T) was harvested before fractionation and soluble tubulin (supernatant, S) and precipitable tubulin (pellet, P) were separated by centrifugation for 1 h at 100 000 g in a Beckman Rotor TLA-100.

Cellular microtubule content was determined by flow cytometry using the method of Morrison et al. [35] with some modifications. Briefly, cells were harvested, rinsed with PBS, centrifuged, washed with MSB and treated for 3 min with MSB containing 0.1 % Triton X-100 and 20 μM taxol. Cells were then fixed in formaldehyde, centrifuged, washed, blocked, incubated for 1 h with FITC-coupled antibodies against α-tubulin and washed. 1500 RFP-positive cells per duplicate sample and condition were analyzed with a FACS ARIA SORP cytometer (Becton Dickinson) and FITC signals plotted with FlowJo software. Control experiments with cells that had been treated with Nocodazole or Taxol showed accuracy.

Growth dynamics of Golgi-derived microtubule were determined essentially as described [5]. Briefly, cells co-transfected with MannII-GFP and RFP or RFP-SOD1 plasmids were treated with nocodazole at 5 DIV (10 μM, 5 h, 37 °C). Nocodazole was then washed out, cultures further incubated for up to 12 min, fixed as indicated above, blocked and counterstained for α-tubulin. After confocal imaging of entire cells, length of Golgi-derived microtubules was determined using Metamorph and ImageJ software respectively.

### Data mining and bioinformatics

Datasets from [19] and [40] were downloaded from the Gene Omnibus database (GSE10953 and GSE46298 respectively). Analysis and visualization of the corresponding Affymetrix CEL files were made using EASANA® software (GenoSplice technology), which is based on the GenoSplice’s FAST DB® annotations [13]. Briefly, microarray data were normalized using RMA and genes were considered significantly differentially expressed when the uncorrected *p*-value from unpaired Student’s *t*-test was lower or equal to 0.05 and fold-change greater or equal to 1.5. Significant KEGG pathways and Gene Ontology terms were retrieved using DAVID.

### Statistical analyses

Each experiment was performed with several biological replicates (see Figure Legends) and repeated at least twice. Data were analysed with Microsoft Excel or GraphPad Prism (GraphPad). Data from two groups showing each Gaussian distribution were analysed with student's *t*-test; otherwise the Mann–Whitney *U* test was used. Data from more than two groups showing each Gaussian distribution and equal variance were analyzed with One Way ANOVA and Tukey posthoc test; otherwise Kruskal-Willis test and Dunn posthoc test were used. Cytometry data were tested for significance with the Chi square test using FlowJo software. Immunofluorescence data were tested for Gaussian distribution by D’Agostino & Pearson test and linear regression and correlation quantified with GraphPad Prism. Subcellular co-localization of SOD1 and MannII-GFP was correlated by computing Pearson coefficients with the image processing package Fiji using six independents assays.

## Abbreviations

ARF, ADP ribosylation factor; BSA: bovine serum albumine; COPI, coat protein complex I; CREB, cAMP response element-binding protein; DIV, day in vitro; DMSO, dimethylsulfoxide; DTT, dithiotreitol; EB3, end-binding protein 3; EDTA, ethylenediaminetetraacetic acid; EGTA, ethylene glycol tetraacetic acid; EZH2, enhancer of zeste homolog 2; FACS, fluorescent-activated cell sorting; GASE, golgi apparatus stress response element; Hepes, 4-(2-hydroxyethyl)-1-piperazineethanesulfonic acid; MannII, mannosidase II; PBS, phosphate buffered saline; Pipes, piperazine-N,N′-bis(2-ethanesulfonic acid); Rab, ras-related in brain; RFP, red fluorescent protein; SDS, sodium dodecyl sulfate; SMA, spinal muscular atrophy; SMN, survival motor neuron; SNARE, soluble N-ethylmaleimide-sensitive- factor attachment receptor; SOD1, superoxide dismutase 1; TBCE, tubulin-binding cofactor E; TFE3, transcription factor binding to IGHM enhancer 3; TPX2, targeting protein for Xklp2; Tris, tris(hydroxymethyl)aminomethane; VAchT, vesicular acetylcholine transporter

## Additional file

Additional file 1:
**Table S1**. Primary and secondary antibodies. **Figure S1**. Golgi structure analyzed by GM130 immunolabeling. **Figure S2**. Subcellular fractionation of spinal cords. **Figure S3**. Flow cytometry analysis of cellular microtubules. **Figure S4**. Labeling of spinal cord sections from SOD1 G85R mice with antibodies against Choline Acetyltransferase (ChAT), Stathmins 1 or 2, Golgi SNARE GS28 and DAPI (nuclei). **Figure S5**. Subcellular localization of Stathmins. (PDF 10467 kb)
