# Supplementary material for: Stathmin 1/2-triggered microtubule loss mediates Golgi fragmentation in mutant SOD1 motor neurons
Source: Mol Neurodegener. 2016 Jun 9;11:43. doi: 10.1186/s13024-016-0111-6 (PMC4899909; doi:10.1186/s13024-016-0111-6)
Supplement: Additional file 1: — Table S1. Primary and secondary antibodies. Figure S1. Golgi structure analyzed by GM130 immunolabeling. Figure S2. Subcellular fractionation of spinal cords. Figure S3. Flow cytometry analysis of cellular microtubules. Figure S4. Labeling of spinal cord sections from SOD1 G85R mice with antibodies against Choline Acetyltransferase (ChAT), Stathmins 1 or 2, Golgi SNARE GS28 and DAPI (nuclei). Figure S5. Subcellular localization of Stathmins. (PDF 10467 kb) [file 13024_2016_111_MOESM1_ESM.pdf]

Supplemental Information

Stathmin 1/2-triggered microtubule loss mediates Golgi fragmentation in mutant SOD1 motor neurons

Sarah Bellouze, Gilbert Baillat, Dorothée Buttigieg, Pierre De La Grange, Catherine Rabouille and Georg Haase

<u>contains :</u>

Supplemental Table S1
Supplemental Figures S1 – S5
Supplemental References

Supplemental Table S1. Primary and secondary antibodies

Primary Antibodies	Dilution IF (WB)	Supplier	Catalog n°/reference
mouse anti-β-actin	1:5000	Sigma	A1978
goat anti-Choline Acetyl Transferase (ChAT)	1:200	Chemicon	Ab144P
mouse anti-Clathrin heavy chain	1:1000	Becton Dickinson	610499
mouse anti-β-COP	(1:1000)	Abcam	Ab6323
rabbit anti-β-COP	(1:1000)	Dr. R. Duden	Duden et al., 1991
mouse anti-GM130	1:300 (1:500)	Becton Dickinson	610823
mouse anti-GS28	1:300 (1:1000)	Becton Dickinson	611185
mouse anti-GS15	1:300 (1:1000)	Becton Dickinson	610961
rabbit anti-L1	(1:2000	Dr. M. Schäfer	Schäfer et al. 2010
rabbit anti-Myc	1 : 400 (1:1000	Cell Signaling	2272
mouse anti-p115	(1:1000)	Becton Dickinson	612260
rabbit anti-Cu/Zn hSOD1	1:1000	Enzo	ADI-SOD-100
rabbit anti-stathmin 1	1:1000 (1:1000)	Dr. A. Sobel	Gavet et al., 1998
rabbit anti-stathmin 2	1:1000 (1:1000)	Dr. A. Sobel	Gavet et al., 1998
rabbit anti-stathmin 3	(1:1000)	Dr. A. Sobel	Gavet et al., 1998
rabbit anti-Sec23	1:1000	Abcam	ab50672
mouse anti-syntaxin 5a	1:10000	Abcam	ab96185
mouse anti-acetylated tubulin	(1:2000)	Sigma	T6793
mouse anti-β_{III}-tubulin	1:10000	Babco	TUJI (MMS-435P)
mouse anti-α-tubulin	1:2000 (1:5000)	Sigma	T9026
rabbit anti-detyr-tubulin	1:2000	Dr. A. Andrieux	Erck et al., 2005
rat anti-Tyr-tubulin	1:2000 (1:50000)	Dr. A. Andrieux	Erck et al., 2005
rat anti-Tyr-tubulin	1:5000	Millipore	YL1/2 (Mab1864)
rabbit anti-VAChT	1:2000	Sigma	V5387
mouse anti-Vti1a	1:1000 (1:1000)	Becton Dickinson	V85620

Secondary antibodies	Dilution	Supplier
goat anti-mouse IgG Alexa 488	1:500	Molecular Probes
goat anti-mouse IgG Cy3	1:2000	Jackson Lab.
goat anti-mouse IgG Alexa 633	1:2000	Molecular Probes
goat anti-rabbit IgG Alexa 488	1:500	Molecular Probes
goat anti-rabbit IgG Cy3	1:2000	Jackson Lab.
goat anti-rabbit IgG Alexa 633	1:2000	Molecular Probes
goat anti-rat IgG Alexa Cy3	1:2000	Molecular Probes

Supplemental Figure S1

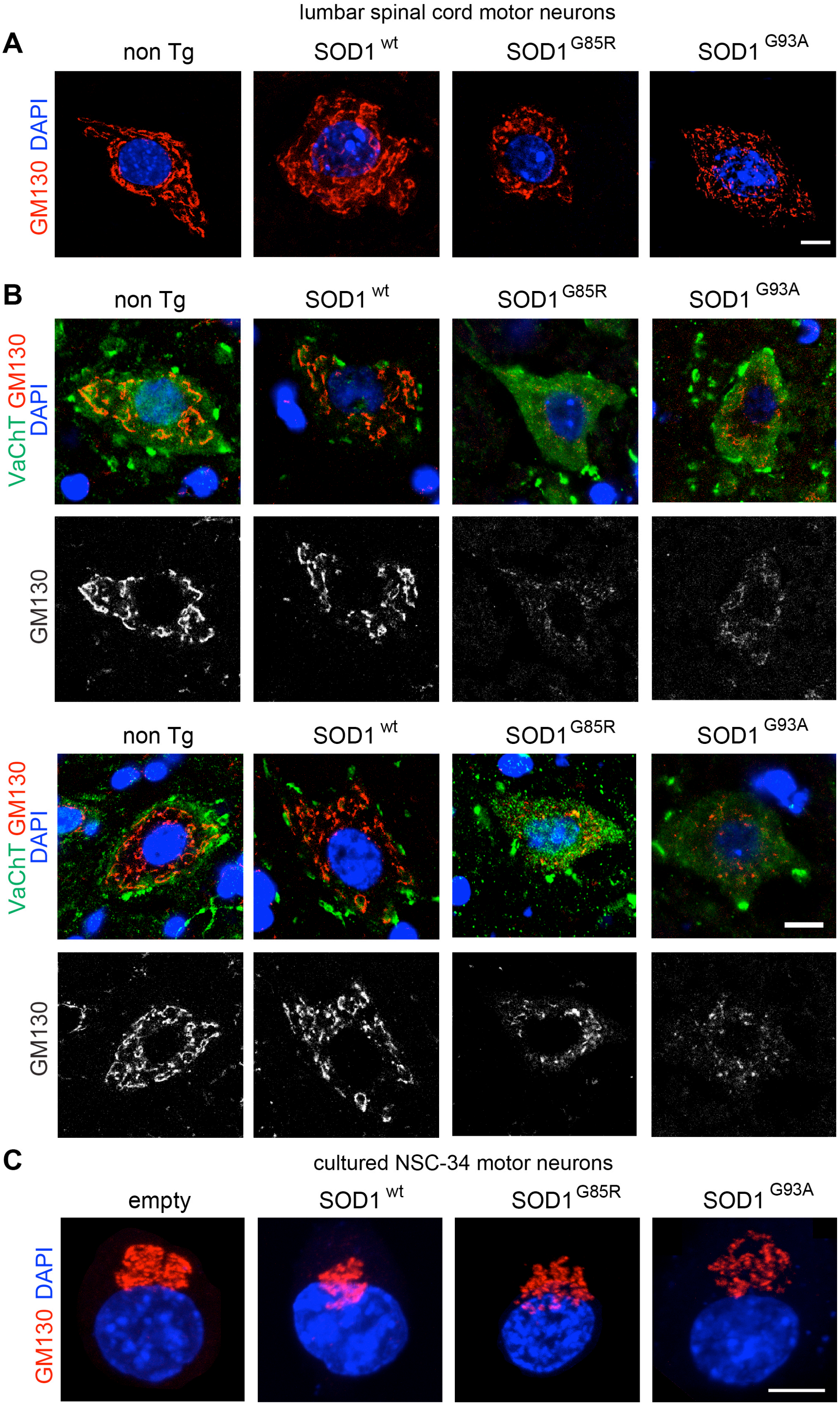

Golgi structure analyzed by GM130 immunolabeling.

A. Single confocal sections at the midplane of the nucleus show apparent reduction of GM130-labelled Golgi area in motor neurons from mice of the indicated genotypes (age 240 days). Scale bar 10 µm.

B. Single confocal sections of lumbar spinal cord from non-transgenic control mice (age 130 days) and transgenic $SOD1^{wt}$ mice (age 180 days) as well as presymptomatic mutant $SOD1^{G85R}$ mice aged 180 days and $SOD1^{G93A}$ mice aged 130 days are identified with the motor neuron marker VAChT. Golgi structure is analyzed with the marker GM130. Black and white images show GM130-labelled Golgi alterations in a fraction of mutant SOD1 motor neurons as compared to controls. Note that non-motor neuronal cells (negative for VaChT) show low GM130 labeling without overt structural Golgi alterations. Scale bar 10 µm.

C. Confocal images showing GM130-labelled Golgi structure in NSC-34 cells transfected for 4 DIV with empty, wildtype SOD1 or mutant SOD1 plasmids.

Supplemental Figure S2

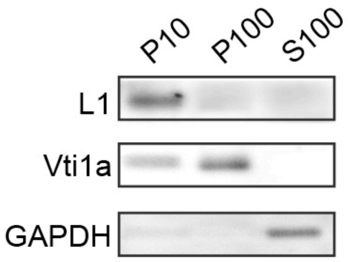

Subcellular fractionation of spinal cords.
Western blot analyses shows purity of subcellular fractions P10, P100 and S100 from lumbar spinal cord attested by markers of membranes (L1), vesicles (Vti1a) and cytosol (GAPDH).

Supplemental Figure S3

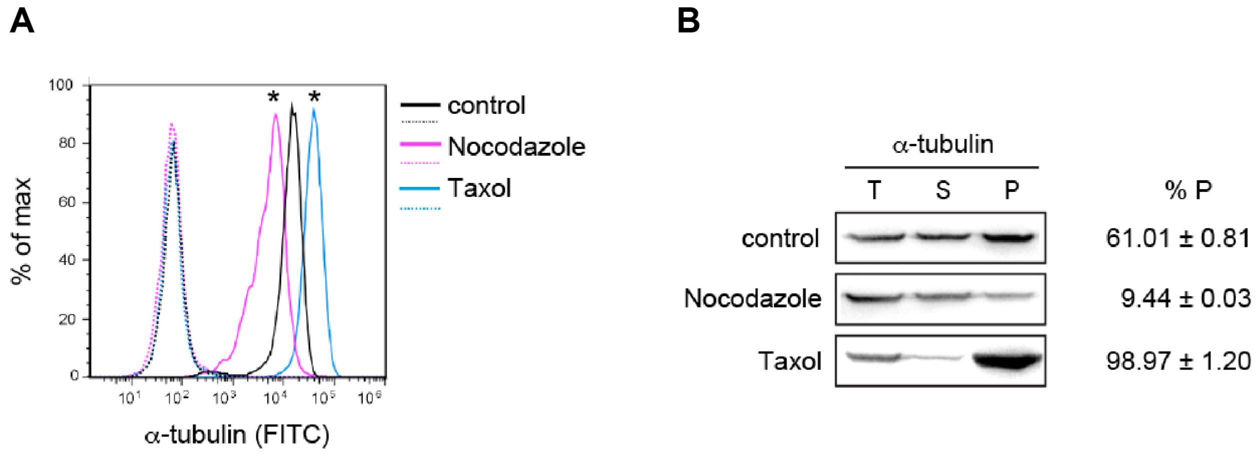

Flow cytometry analysis of cellular microtubules.

A. Flow cytometry. Diagram showing the cellular content in polymerized α-tubulin in NSC-34 cells after extraction of soluble proteins, incubation with anti-α-tubulin-FITC antibodies and flow cytometry analysis. Solid lines correspond to cells that had been treated with mock (control), Nocodazole or Taxol. Dotted lines correspond to cells incubated without antibodies. Median fluorescence values and their statistical difference with respect to control by chi-test : control 12.800, Nocodazole 4.770, T(x)=5.616 (*); Taxol 34.900, T(x)=5.730 (*).

B. Subcellular fractionation. Immunoblots showing total α-tubulin (T), soluble α-tubulin (S) and polymerized α-tubulin in NSC-34 motor neurons treated with mock (control), Nocodazole or Taxol. The fraction of polymerized tubulin (% P representing P/(S+P)) is indicated on the right. Differences are statistically significant by Mann-Whitney test.

Supplemental Figure S4

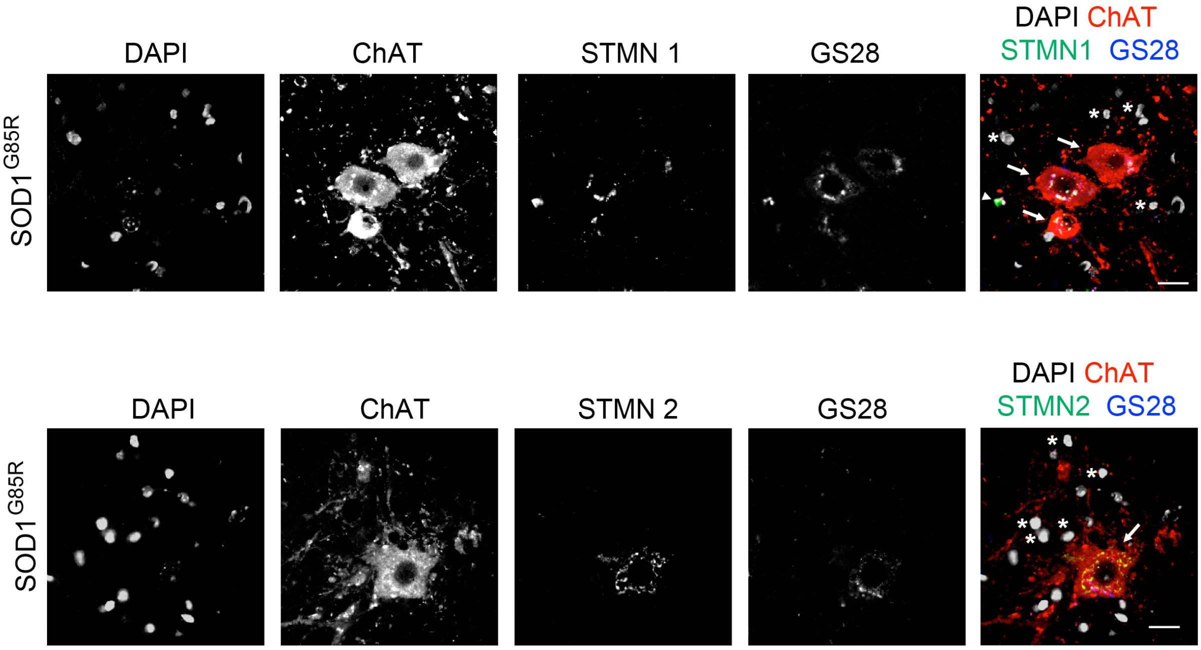

Labeling of spinal cord sections from SOD1 G85R mice with antibodies against Choline Acetyltransferase (ChAT), Stathmins 1 or 2, Golgi SNARE GS28 and DAPI (nuclei).

Motor neurons were identified by ChAT immunoreactivity and large, faintly DAPI-stained, nuclei, as indicated by arrows on merged images. Non-motor neuronal cells were identified by negative ChAT and positive DAPI labeling ; some of them are indicated by asterisks.

A. Confocal images show up-regulation of both Stathmin 1 and GS28 in motor neurons. Expression of Stathmin-1 and GS28 in other cell types of mutant SOD1 G85R spinal cord is below threshold. For quantitative immunofluorescence analyses non-saturated images were used and partially sectioned motor neurons (arrowhead) were excluded.

B. Confocal images showing up-regulation of Stathmin 2 and GS28 in a motor neuron.

Scale bar 20 μm.

Supplemental Figure S5

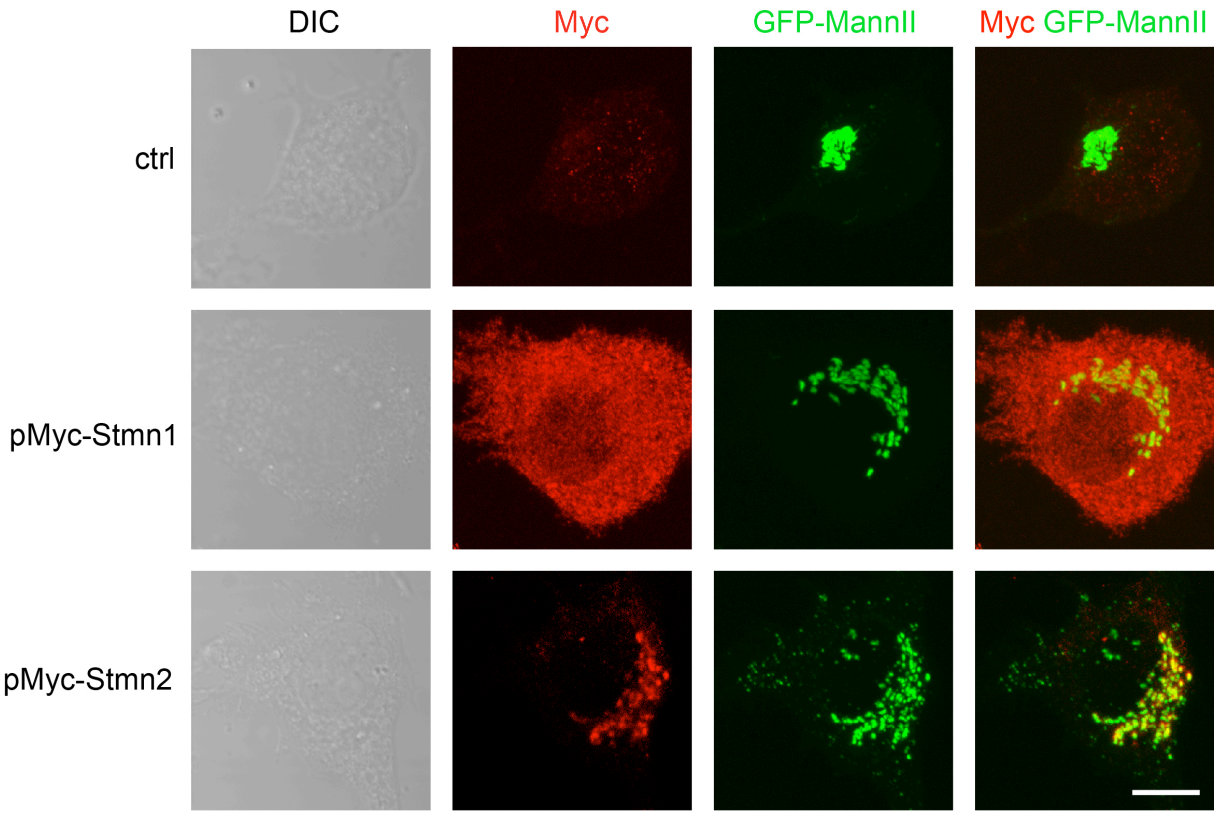

Subcellular localization of Stathmins.
Confocal images showing NSC-34 motor neurons transfected with Myc-tagged forms of Stathmin 1 or Stathmin 2 or control, and labeled with anti-Myc antibodies and the co-transfected marker MannosidaseII-GFP (MannII-GFP). Differential interphase contrast (DIC) images indicate cell contours. While Stathmin 1 is mainly cytosolic, Stathmin 2 localizes to the Golgi. Overexpression of Stathmin 1 or 2 causes Golgi disruption. Scale bar 10 μm.

Supplemental References

Duden R, Griffiths G, Frank R, Argos P, Kreis TE (1991) Beta-COP, a 110 kd protein associated with non-clathrin-coated vesicles and the Golgi complex, shows homology to beta-adaptin. *Cell* **64:** 649-665

Erck C, Peris L, Andrieux A, Meissirel C, Gruber AD, Vernet M, Schweitzer A, Saoudi Y, Pointu H, Bosc C, Salin PA, Job D, Wehland J (2005) A vital role of tubulin-tyrosine-ligase for neuronal organization. *Proceedings of the National Academy of Sciences of the United States of America* **102:** 7853-7858

de la Grange P, Dutertre M, Correa M, Auboeuf D (2007) A new advance in alternative splicing databases: from catalogue to detailed analysis of regulation of expression and function of human alternative splicing variants. *BMC Bioinformatics* **8:** 180

de la Grange P, Dutertre M, Martin N, Auboeuf D (2005) FAST DB: a website resource for the study of the expression regulation of human gene products. *Nucleic Acids Res* **33:** 4276-4284

Ferraiuolo L, Heath PR, Holden H, Kasher P, Kirby J, Shaw PJ (2007) Microarray analysis of the cellular pathways involved in the adaptation to and progression of motor neuron injury in the SOD1 G93A mouse model of familial ALS. *The Journal of neuroscience* **27:** 9201-9219

Gavet O, Ozon S, Manceau V, Lawler S, Curmi P, Sobel A (1998) The stathmin phosphoprotein family: intracellular localization and effects on the microtubule network. *J Cell Sci* **111:** 3333-3346

Nardo G, Iennaco R, Fusi N, Heath PR, Marino M, Trolese MC, Ferraiuolo L, Lawrence N, Shaw PJ, Bendotti C (2013) Transcriptomic indices of fast and slow disease progression in two mouse models of amyotrophic lateral sclerosis. *Brain* **136:** 3305-3332

Perrin FE, Boisset G, Docquier M, Schaad O, Descombes P, Kato AC (2005) No widespread induction of cell death genes occurs in pure motoneurons in an amyotrophic lateral sclerosis mouse model. *Hum Mol Genet* **14:** 3309-3320

Schäfer MK, Nam YC, Moumen A, Keglowich L, Bouché E, Küffner M, Bock HH, Rathjen FG, Raoul C, Frotscher M (2010) L1 syndrome mutations impair neuronal L1 function at different levels by divergent mechanisms. *Neurobiol Dis* **40**: 222-37. doi: 10.1016/j.nbd.2010.05.029
